# Working memory, attention, and salience in active inference

**DOI:** 10.1038/s41598-017-15249-0

**Published:** 2017-11-07

**Authors:** Thomas Parr, Karl J Friston

**Affiliations:** Wellcome Trust Centre for Neuroimaging, Institute of Neurology, University College London, WC1N 3BG London, UK

## Abstract

The psychological concepts of working memory and attention are widely used in the cognitive and neuroscientific literatures. Perhaps because of the interdisciplinary appeal of these concepts, the same terms are often used to mean very different things. Drawing on recent advances in theoretical neurobiology, this paper tries to highlight the correspondence between these established psychological constructs and the formal processes implicit in mathematical descriptions of brain function. Here, we consider attention and salience from the perspective offered by active inference. Using variational principles and simulations, we use active inference to demonstrate how attention and salience can be disambiguated in terms of message passing between populations of neurons in cortical and subcortical structures. In brief, we suggest that salience is something that is afforded to actions that realise epistemic affordance, while attention *per se* is afforded to precise sensory evidence – or beliefs about the causes of sensations.

## Introduction

In order to interact optimally with our environment, we need to correctly infer the state of the world around us. Given only the data obtained from sensory epithelia, such as the retina, any organism needs to be able to form accurate beliefs about the causes of these data. If those beliefs are correct, the organism will be able to act so that it searches out or creates surroundings sympathetic to its continued survival. The construction of beliefs about a sensory scene has been compared to the scientific endeavour^1^, in which hypotheses are made concerning the nature of the world, and experiments are made to test the predictions of the hypotheses. These perceptual experiments should resolve the uncertainty about which hypothesis best describes incoming (experimental or sensory) data. In the context of the visual system, saccadic eye movements can be thought of as experiments^2^. By strategically foveating specific regions of visual space, animals can garner evidence to support or refute their beliefs about the hidden states generating a visual scene. If perception is considered in terms of this metaphor, it becomes clear that there is a need for a form of memory to take previous sensory information into account – when planning future saccades. This motivates the link made below between working memory and evidence accumulation. The process of planning future saccades corresponds well with some definitions of attention^3,4^, but contrasts with others^5–7^. It is argued here that attention is frequently used to refer to two very different phenomena. One relates to salience, and is fundamentally a property of action plans and epistemic affordance. The other involves the biasing of inference towards sensory channels providing precise information. The first part of this paper will consider how various accounts of these cognitive processes can be reconciled through recent theoretical advances, and the second will demonstrate that characteristic behavioural and physiological manifestations of each can be reproduced in silico by appealing to the principles of active inference. To do this, we employ simulations of an agent that conforms to these principles. Related simulations have previously been used to illustrate a range of cognitive phenomena^8–12^.

## Anatomy

The neuroanatomy underlying beliefs about the environment is crucial for a biologically grounded understanding of the processes by which these beliefs are formed and evolve. Three important aspects of the anatomy will be covered here, as they relate closely to attention and working memory. First the anatomy involved in selecting saccadic eye-movements will be considered. This is important for the concept of salience in attention, as the eyes are directed towards salient locations in visual space^9^. It is also very important for working memory, as the choice of salient location depends on previously sampled locations. Second is a discussion of the hierarchical structure of the cerebral cortex. The interactions between different areas will be seen to be crucial to attention^7^, while the properties of each level relative to each other provide important hints as to the biological basis of working memory. Thirdly, subcortical structures will be considered, in terms of both salience and hierarchy.

### Saccadic eye-movements

**Figure 1:**
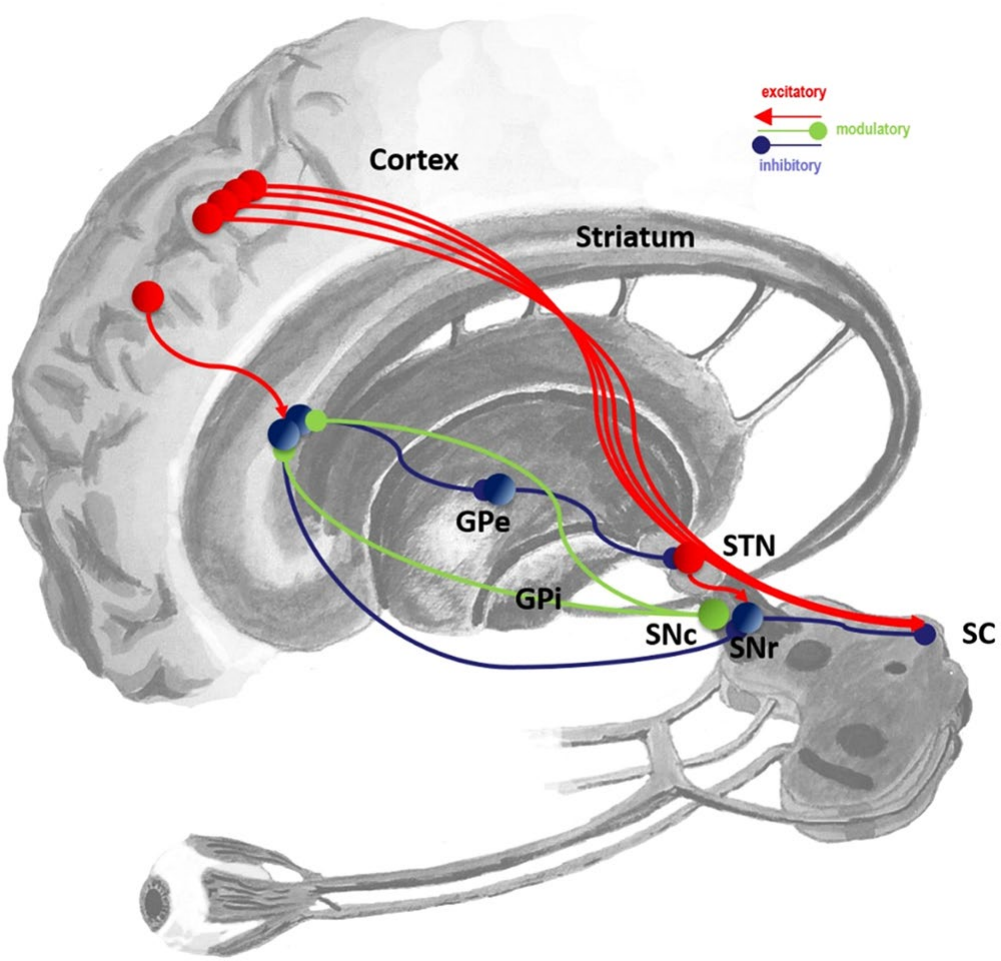
Neuroanatomy of eye-movement control This figure illustrates the anatomy of the inputs the superior colliculus (SC) receives from the basal ganglia, noting that there is additionally a convergence of input from a diverse set of cortical regions. For clarity, other inputs to the SC have been omitted, but it should be remembered that the vestibular and cerebellar projections to the midbrain are vitally important in some aspects of saccadic control. The basal ganglia influence the SC via the substantia nigra pars reticulata (SNr), which is the point of convergence between the direct, indirect, and hyperdirect pathways. All three of these pathways are shown, with some simplifications. The direct pathway is from cortex to striatum to the SNr. The indirect is from cortex to striatum to the external segment of the globus pallidus (GPe) to the subthalamic nucleus (STN) to the SNr. The hyperdirect is from cortex to STN to SNr. The substantia nigra pars compacta (SNc) and the ventral tegmental area (VTA - not shown) of the midbrain provide a dopaminergic input to the striatum. This schematic is based on descriptions in^91^ and adapted from^134^.

The superior colliculus is a key site for the control of saccadic eye-movements. It is found in the midbrain, at the level of the oculomotor nucleus (cranial nerve III). Each layer of the superior colliculus contains a topographically organised map. The most superficial of these is a map of visual space in retinal coordinates^13^, making use of the direct input from the retina. An intermediate layer is a motor map^14^, determining saccadic target locations. The deepest layer has a multisensory map^15,16^, incorporating somatotopic information. A number of cortical areas project to the superior colliculus^17–19^, including the frontal eye fields^20^, lateral intraparietal cortex^21^, and the supplementary eye fields^22^ (Fig. 1). This convergence of input can be thought of as identifying potential saccadic targets. An inhibitory input to the superior colliculus arises from the substantia nigra pars reticulata (SNr)^23^, a GABAergic output nucleus of the basal ganglia. Disruption of this nigro-collicular connection^24^, or of the activity of SNr neurons^25^, impairs the maintenance of fixation, and increases the frequency of spontaneous saccades. These findings are consistent with the basal ganglia providing a tonic inhibitory output to the superior colliculus, which can be relieved to allow phasic disinhibition, leading to a saccade.

The SNr receives input from the striatum and the subthalamic nucleus (STN), which carry information through the direct and indirect (or hyperdirect) pathways respectively^26^. Activity in the former pathway disinhibits the targets of the SNr projection neurons, while the latter increases the inhibition^27^. The relative activity in the two pathways is modulated by dopaminergic projections to the striatum^28^. These enhance the activity of D1 receptor expressing striatal medium spiny neurons (MSNs) which feed into the direct pathway, and suppress the activity of D2 expressing MSNs, which project to the external part of the globus pallidus which in turn projects to the STN as part of the indirect pathway.

Based on this anatomy, it seems that dopamine should influence when and where a saccade takes place. Infusing MPTP, a substance toxic to dopaminergic axons, into the caudate nucleus unilaterally has little effect on visually guided saccades. However, when memory guided saccades are performed, there is a biasing of saccades towards locations in the ipsilateral visual field^29^. This is also true of the area scanned by spontaneous saccades^30^. Parkinson’s disease, which involves degeneration of the dopaminergic midbrain, also impairs memory guided saccades^31^. This implicates the basal ganglia in both working memory and salience based attentional processes.

### Cortical hierarchies

**Figure 2:**
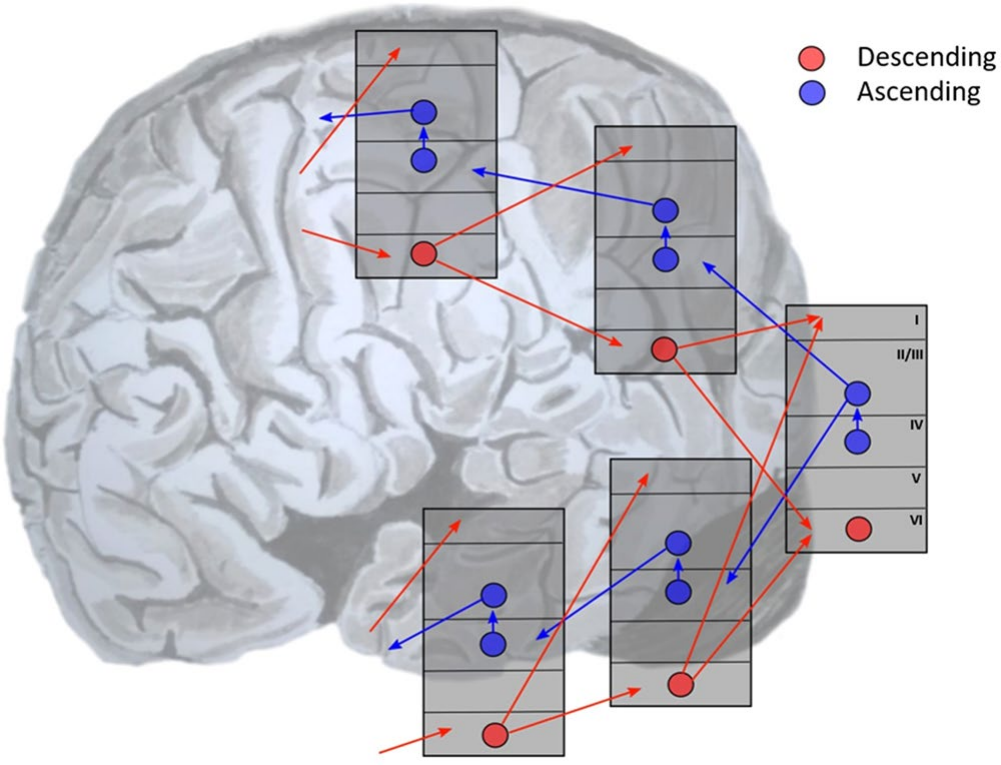
Cortical hierarchies There is a systematic, stereotyped pattern of laminar-specific connectivity in the cerebral cortex. Layer IV receives ascending (extrinsic) input, and has (intrinsic) connections to more superficial layers (II/III) in the same region. This layer provides (extrinsic) projections to layer IV in higher cortical areas and (intrinsic) connections to deep layer VI. Layer VI projects to both deep and superficial layers in lower regions, and this provides a pattern of descending (extrinsic) connections. These patterns of (extrinsic) connectivity can be used to map out hierarchies in the brain. The hierarchies in the two streams shown here ascend from the right of the image to the left. This schematic is based on the descriptions in^36^.

One of the fundamental principles of cerebral cortical anatomy is that regions are arranged according to a common hierarchy^32,33^. This can be defined both structurally and functionally. Structurally, there are stereotyped patterns of laminar connectivity between cortical regions (i.e., extrinsic connectivity) in both the ascending and descending directions^34–36^. Ascending connections target layer IV that provides within region (i.e., intrinsic connectivity) input to superficial layers. These layers are the source of further ascending (extrinsic) connections to higher areas and forward (intrinsic) interlaminar projections to deep layers. Descending extrinsic connections originate in layer VI, and project to both deep and superficial layers of the lower cortical area. These are illustrated in Fig. 2, which shows two hierarchical streams originating from the occipital cortex. Note that (subcortical) projections to both the basal ganglia and the SC arise from layer V^18,36^. In predictive coding theories of attention, based on gain control, it is the (source of) ascending connections between regions that are thought to be modulated by attention^7^.

Functional specialisation depends on the receptive fields of neurons in a particular area^33^. Three properties are of interest here. The first is the complexity of the stimulus which maximally excites neurons in each region. This complexity increases as the hierarchy is ascended^37^, with early visual areas responsive to oriented bars^38^, or contours^39,40^, but higher areas responding selectively to, for example, faces^41^. The second and third functional properties which vary are the spatial^42,43^ and temporal^44–47^ receptive field sizes respectively. Both are larger in higher areas. Structural and functional attributions of cortical areas all converge on a common architecture; namely a cortical hierarchy, reinforcing the notion that this hierarchical form is a defining principle of cortical organisation. It is likely that this form manifests a fundamental set of computations, central to brain function.

The increasing size of temporal receptive field is particularly interesting here. Representations in higher regions typically evolve over a longer time scale than lower levels. This means they can transcend the timescales of (transient) sensory sampling. A ubiquitous example of this is delay period activity (which persists once a stimulus has been removed), reminiscent of working memory^48^. Slowly evolving representations can provide the context for faster changes in the lower area, influencing the interpretation of a given stimulus. Additionally, as the higher level representation accumulates information from previous sensory samples, it informs actions over a longer time scale than the lower level. This might explain why cortical areas considered to be high in the hierarchy, such as the dorsolateral prefrontal cortex^32^, have been heavily implicated in working memory^49^. In the later sections, it will be argued that working memory is a process of evidence accumulation, which occurs at multiple temporal scales^44,46^ in order to inform action choices. The functional link between these hierarchical levels and action suggests it is worth considering the anatomical links between high levels of deep cortical hierarchies and the important subcortical structures involved in action planning.

### Subcortical hierarchies

**Figure 3:**
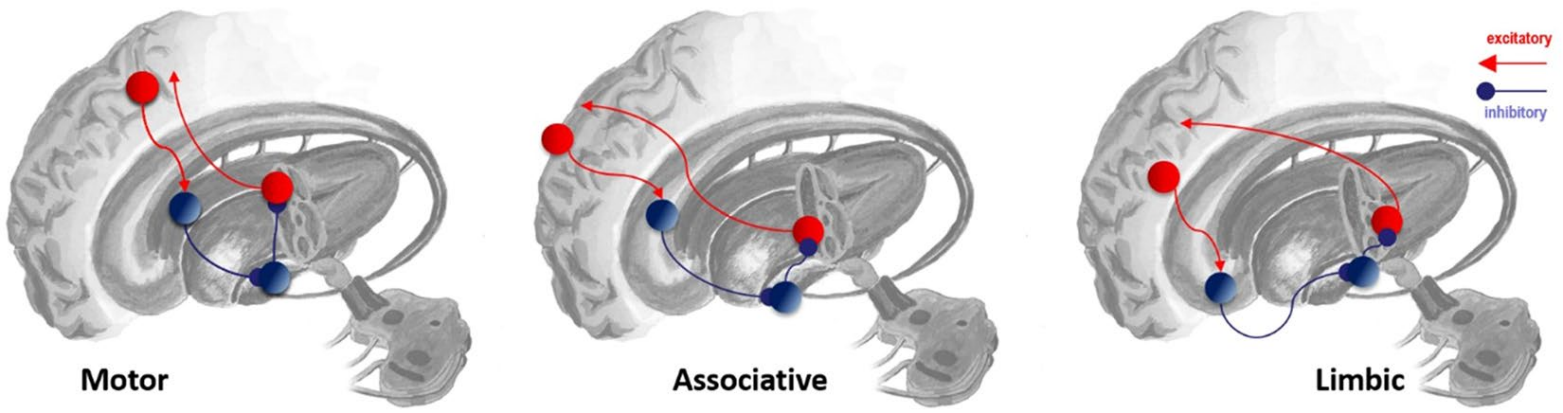
Parallel cortico-subcortical loops Cortical areas target particular regions of the basal ganglia. This cortical input is the start of a loop through the basal ganglia, back to the same cortical area via the thalamus. For simplicity, only the direct pathway through the basal ganglia is shown in these figures. Connections from the dopaminergic midbrain are not shown, but these target the caudate and putamen. These schematics are based on^61^.

Figure 3 shows how the cortical hierarchy is replicated in the basal ganglia. This set of subcortical structures has been implicated in working memory processes in a number of experimental^50–54^ and theoretical^55–58^ studies. The main input nucleus of the basal ganglia is the striatum (a structure which includes both the caudate and putamen). This structure receives cortical input in a topographically organised manner, with higher cortical regions projecting to the more ventral part, and lower cortical regions projecting to the more dorsolateral parts^59^. Starting from the striatum, there is a set of parallel loops^59–61^, each corresponding to different cortical hierarchical levels, which eventually project back to the cortical region of their origin, via the thalamus. The functions of these loops have been primarily addressed in the motor domain, as the basal ganglia are heavily implicated in planning actions. However basal ganglia outputs also target sensory^62^ and prefrontal^63^ regions. That there are distinct loops from different hierarchical levels suggests action planning (or policy selection) takes place at differing levels of abstraction and over different time courses. Hierarchical policy planning would require representations of the proximate past and future at each level^64^, consistent with the notion of a working memory system organised in a temporal hierarchy.

Clinical observations further endorse this notion of behavioural planning over different time scales, but are dependent on an additional expression of topography in this system. The dopaminergic midbrain includes both the SNc and the VTA, and these both target (among other areas) the striatum. Whereas the SNc targets more dorsolateral aspects of the striatum, progressively more ventral areas have increasing dopaminergic input from the VTA^59^. This means that disease processes which affect the SNc, such as Parkinson’s disease, should result in deficits in motor planning at a lower hierarchical level, and therefore over a faster time scale. In contrast disorders involving the VTA should cause disordered behaviour which manifests over a much longer time scale. It is the more ventral parts of this system which are thought to be implicated in disorders such as schizophrenia^65,66^ or addiction^67^. Whereas in Parkinson’s disease, the policy deficit is apparent even when initiating simple motor actions, the other two disorders involve no impairment in a simple motor act, but do involve behavioural abnormalities over a longer time period.

The planning of actions (or policy evaluation) in the basal ganglia has a particular relevance for attention, in the “salience” sense of the term. Salience can be viewed as a property of locations that are potential saccadic targets. More salient locations are those which are more likely to be foveated. It follows that evaluating salience must then correspond to evaluating competing choices of saccade. As is clear from the section above, the basal ganglia are well situated anatomically to perform this function. The anatomy here harmonises well with models which implicate the basal ganglia explicitly in action selection, including a dopaminergic modulation of this process^68^. Additionally, measured short latency visual responses in dopaminergic neurons following disinhibition of the superior colliculus^69^ suggest an important role in selection of eye movements in particular^70^.

In what follows, we will pick up on the key functional anatomic features surveyed above – and show how the implicit (cortico cortical and cortico-subcortical) hierarchical message passing conforms closely to what would be predicted by active inference formulations of brain function. We first overview the computational architectures implied by active inference and then use simulations to reproduce some key (behavioural and neuronal) responses that had been observed in empirical studies of working memory and attention.

## Active inference

This section provides an overview of the theory behind active inference, in addition to a description of the generic structure of a Markov decision process (MDP) models used to simulate the evaluation and selection of actions. Readers familiar with this material are invited to skip the following sections, until the discussion of the form working memory and attention take in this formulation.

### Motivating principles

Active inference formalises some of the, colloquially described, notions from above. It is based on the tautological notion that an adaptive agent in general, and a living organism in particular, should avoid crossing phase boundaries if they are to continue to exist in the same form^71^. An example of a phase boundary, which illustrates this point clearly, is a cliff edge. For a flightless organism, structural integrity depends on keeping to one side of this boundary. In order to avoid such boundaries, it is necessary for an agent to minimise the dispersion of observable outcomes (e.g., where it is in relation to the edge). The information an agent has access to about the states of the world (and itself) is contained in observable sensory states, and it is the dispersion of these the agent must limit. Mathematically, the dispersion of observable states $\tilde{\rm{o}}$ can be expressed as their Shannon entropy.

1 $$H[P({\tilde{\rm{o}}})]=-{E}_{P({\tilde{\rm{o}}})}[\,{\rm{ln}}\,P({\tilde{\rm{o}}})]$$

The notation ${\tilde{\rm{o}}}$ means (*o*_1,_ *o*_2,_ *o*_3…_ *o*_*T*_)^*T*^. *E*_p_[·] means the expectation with respect to the distribution *P*. Note that the contribution made to this quantity by each outcome is greater the more unlikely that outcome is. This means a system which occupies a small number of states, each of high probability, has a low entropy. Under ergodic assumptions (that is, averaging over sufficient time, the behaviour of the system should match that found from averaging over all of the states in the system), the average of the negative log probability of outcomes (surprise) should converge to the entropy^72^. This means that organisms must minimise their surprise over time. Outcomes are generated by hidden states, $\widetilde{s}$, in the environment, and are influenced by the policies, *π*, an agent pursues. The marginal probability of outcomes according to a generic generative model is then:

2 $$P({\tilde{\rm{o}}})=\sum _{\widetilde{s},\pi }P({\tilde{\rm{o}}},\widetilde{s},\pi )$$

The sum is replaced by an integral in continuous state-space generative models. A generative model expresses the beliefs an agent has about the causal structure of their world. One form this model can take is that of a Markov decision process, and this will be described in more detail below. The summation above is often intractable, so the minimisation of surprise cannot be performed directly. Instead an upper bound on surprise is used. This is found by noting that multiplying and dividing the joint probability above by an arbitrary function $Q(\widetilde{s},\pi )$ does not change its value. This means the surprise can be expressed:

3 $$-{\rm{ln}}\,P({\tilde{\rm{o}}})=-{\rm{ln}}\,{\sum }_{\widetilde{s},\pi }\frac{P({\tilde{\rm{o}}},\widetilde{s},\pi )Q(\widetilde{s},\pi )}{Q(\widetilde{s},\pi )}=-{\rm{ln}}\,{E}_{Q(\widetilde{s},\pi )}[\frac{P({\tilde{\rm{o}}},\widetilde{s},\pi )}{Q(\widetilde{s},\pi )}]$$

Appealing to Jensen’s inequality, the expectation of a logarithm is always less than the logarithm of an expectation. This is due to the curvature of a logarithmic function, as the former will lie beneath the curve, and the latter is, by definition, on the curve. Using this fact, and moving the expectation outside the logarithm of the expression above will result in the inequality:

4 $$-{\rm{ln}}\,P({\tilde{\rm{o}}})=-{\rm{ln}}\,{E}_{Q(\widetilde{s},\pi )}[\frac{P({\tilde{\rm{o}}},\widetilde{s},\pi )}{Q(\widetilde{s},\pi )}]\le -{E}_{Q(\widetilde{s},\pi )}[{\rm{ln}}\,\frac{P({\tilde{\rm{o}}},\widetilde{s},\pi )}{Q(\widetilde{s},\pi )}]=F$$

The term on the right hand side of the inequality is therefore an upper bound on the surprise. This is known as the free energy^73,74^. If the bound is tight, minimisation of free energy minimises surprise. The arbitrary distribution is factorised according to something called a mean field approximation. This allows it to be expressed as the product of distributions for states at particular times given policies, and of the policies. The position invariance of some representations at higher levels of the ventral visual pathway is reminiscent of such a factorisation of states, as it suggests “what” can be represented independently of “where”^75,76^ and of “when”^77^.

To preclude surprises (e.g., falling over the cliff edge) it is therefore sufficient to minimise free energy which, incidentally, is the same as maximising the marginal likelihood or Bayesian model evidence $P({\tilde{\rm{o}}})$. This equivalence offers a deep connection between self organisation in living systems and notions like the Bayesian brain^78–81^. Minimisation of the free energy can then proceed by optimising the factors or marginals of the arbitrary distribution (which is the approximate posterior distribution). In general, the minimum for each factor assuming all others are fixed can be found by differentiating the free energy with respect to that factor, while constraining the marginal distribution to sum to one (e.g. using a Lagrange multiplier). Setting the result to zero and rearranging gives^82,83^ (for the distribution over *s*_*t*_|*π*):

5 $${\rm{l}}{\rm{n}}\,{Q}^{\ast }\,({s}_{t}|\pi )={E}_{Q(/{s}_{t}|\pi )}[{\rm{l}}{\rm{n}}\,P(\mathop{o}\limits^{ \sim },\mathop{s}\limits^{ \sim },\pi )]\,-\,{\rm{l}}{\rm{n}}\,Z$$

Here the notation *Q*(/*s*_*t*_|*π*) means the product of all factors of the approximate posterior with the exception of the distribution over *s*_*t*_|*π*. *Z* is a constant, called a partition function in physics. To find the appropriate belief updates, the generative model must be defined. It is here that things like predictive coding^84,85^ and Bayesian decision theory with Markov decision processes (MDPs)^86^ part ways. The former schemes use models with continuous state spaces, whereas the latter schemes deal with discrete or categorical states. Happily, both can be formulated as a gradient descent on variational free energy, which brings *Q*(*s*_*t*_|*π*) towards Q*(*s*_*t*_|*π*). Here, our focus will be on the MDP formulation, but it will be useful later on to remember that the two are closely related.

### Basic structure of the MDP

**Figure 4:**
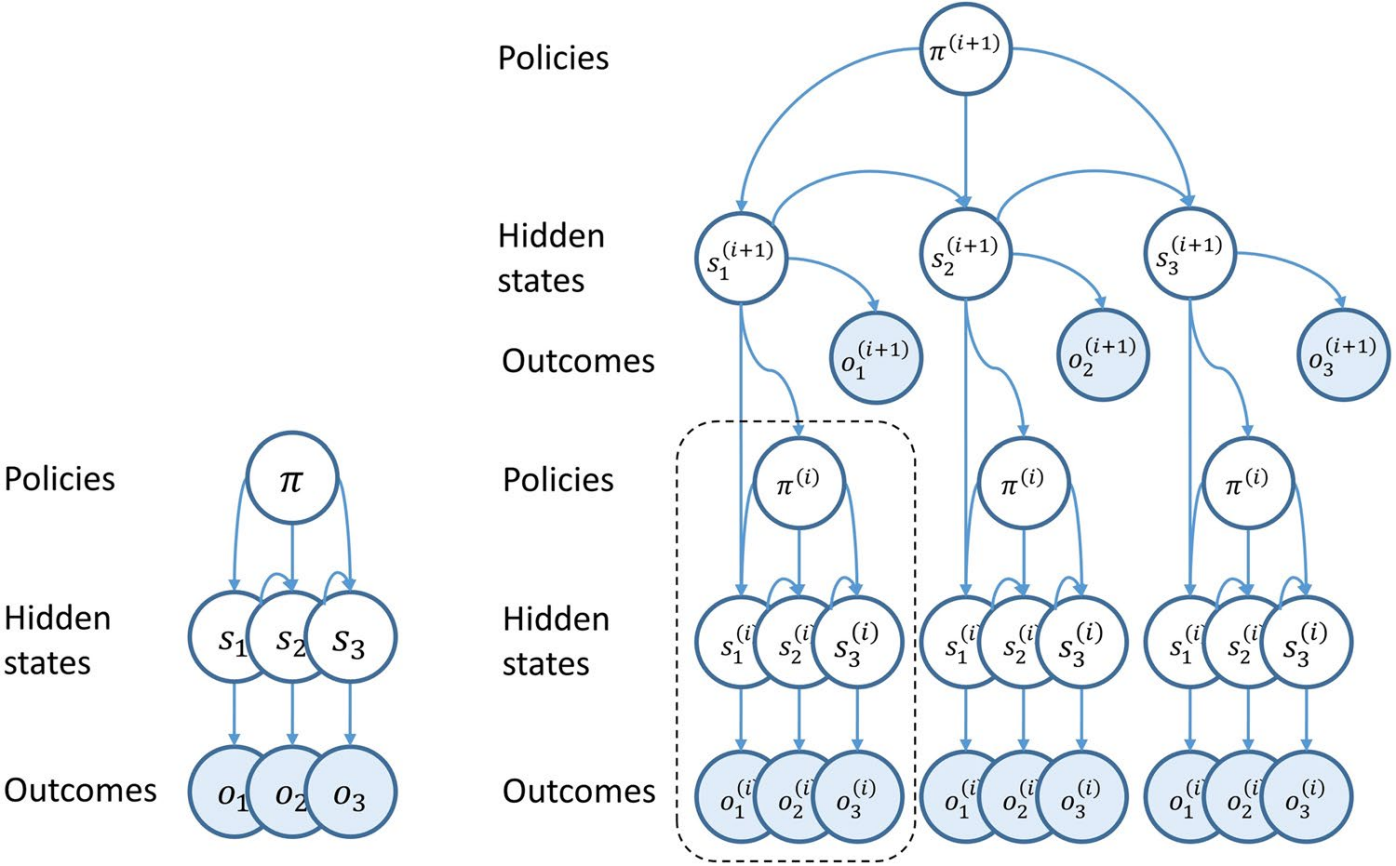
–MDP generative models *On the left* is the basic structure of the MDP generative model, and on the right is a generalisation to a deep temporal hierarchical model. Arrows indicate conditional dependencies so that, for example, the arrow from *s*_3_ to *o*_3_ on the left indicates that ${o}_{3}$ depends on *s*_3_ (and only on *s*_3_ directly) according to some probability $P({o}_{3}| {s}_{3})$. The absence of an arrow between two variables indicates conditional independence, which allows factorisation of the joint probability of the generative model. Note that the hierarchical model *on the right* includes multiple state transitions at a given level (where level is indicated by bracketed superscripts) for each single state transition at the level above. The hierarchical model introduces additional dependencies, which include making both policy and the first hidden state at one level dependent on the hidden state at the level above.

The MDP generative model is defined so that outcomes depend only on hidden states. This relationship is described by a matrix **A**, where ${{\bf{A}}}_{ij}=P({o}_{t}=i| {s}_{t}=j)$. Hidden states depend on previous states, and on policies. This dependency is described by the probability transition matrix **B**, where ${{\bf{B}}}_{ij}(u)=P({s}_{t+1}=i| {s}_{t}=j,\pi (t)=u)$. **C** defines prior beliefs about outcomes, with components representing $P({o}_{t})$, and **D** encodes the beliefs about initial states^9^. These conditional probabilities are represented graphically in the Bayesian network on the left of Fig. 4. Here, an arrow from one variable to another indicates that the second variable is dependent on the first (see caption for details). The matrices described above define these conditional probabilities.

To ensure agents act to minimise free energy over time, they should choose the policies which minimise their expected free energy, *G*(*π*). This imperative is included in the model as a prior belief over policies $P(\pi )=\sigma (-\gamma \cdot G(\pi ))$, where *σ*(·) is a softmax function that takes values between zero and one; making this a proper probability distribution. *γ* is an inverse temperature parameter, which acts as a precision over policies. Agents therefore believe that they will pursue policies (sequences of actions) that minimise their expected free energy.

The free energy was defined above as:

6 $$F=-{E}_{Q(\widetilde{s},\pi )}[{\rm{ln}}\,P({\tilde{o}},\widetilde{s},\pi )\,-\,{\rm{ln}}\,Q(\widetilde{s},\pi )]$$

By analogy with this, but conditioning on the policy, expected free energy at a particular time point becomes:

7 $$G(\pi ,\tau )=-{E}_{Q({s}_{\tau }| \pi )P({o}_{\tau }| {s}_{\tau })}[{\rm{ln}}\,P({o}_{\tau },{s}_{\tau }| \pi )\,-\,{\rm{ln}}\,Q({s}_{\tau }| \pi )]$$

Which can then be summed over future time to give $G(\pi )={\sum }_{t\ge \tau }G(\pi ,\tau )$, the total expected free energy. A rearrangement of this equation provides an interesting interpretation of this quantity:

8 $$G(\pi ,\tau )=-{E}_{Q({s}_{\tau }| \pi )P({o}_{\tau }| {s}_{\tau })}[{\rm{ln}}\,P({o}_{\tau })]+{E}_{Q({s}_{\tau }| \pi )P({o}_{\tau }| {s}_{\tau })}[{\rm{ln}}\,Q({s}_{\tau }| \pi )\,-\,{\rm{ln}}\,P({s}_{\tau }| {o}_{\tau },\pi )]$$

The first term here is the expected surprise, and this indicates that policies should be selected which the agent believes will fulfil its prior beliefs about outcomes in the future. This corresponds to exploitation of an advantageous policy given current knowledge as – based on the current distribution *Q*(*s*_*t*_|*π*)– the agent will act to minimise its expected surprise or cost encoded by **C**. The second term, in contrast, will be minimised by observations that bring the approximate posterior distribution close to the true posterior. Technically, this term is an expected divergence or Bayesian surprise: see below and^87^. This corresponds to information gain or mutual information between future outcomes and the hidden states that cause them. If new observations cease to be informative, this epistemic term must have reached its minimum. Reducing this component of expected surprise corresponds to uncertainty reduction, or exploration. In short, the drives towards pursuing a particular course of action, such as selecting a saccade target, can be decomposed into the fulfilment of prior beliefs about outcomes and the resolution of uncertainty about states of the world generating those outcomes. In other words, policies the agent beliefs it will pursue have both pragmatic (surprise avoiding) and epistemic (uncertainty reducing) affordance.

### Message passing

Once the MDP has been defined in terms of the **A**, **B**, **C** and **D** matrices described above, the messages which are passed between neuronal representations of hidden states can be derived using the equation for ${\rm{l}}{\rm{n}}\,{Q}^{\ast }({s}_{t}|\pi )$ above. For a given state *s*_*t*_, only a small number of other states directly influence – or are influenced by – it. Given this set of states (the Markov blanket), *s*_*t*_ is conditionally independent of all other states. By absorbing the factors of $P({\tilde{\rm{o}}},\widetilde{s},\pi )$ that do not involve interactions between *s*_*t*_ and its Markov blanket into the constant Z, the equation can be simplified to give^88,89^:

9 $${\rm{l}}{\rm{n}}\,{Q}^{\ast }\,({s}_{t}|\pi )={E}_{Q(/{s}_{t}|\pi )}[{\rm{l}}{\rm{n}}\,P({s}_{t}|{s}_{t-1},\pi )+\,{\rm{l}}{\rm{n}}\,P({s}_{t+1}|{s}_{t},\pi )+\,{\rm{l}}{\rm{n}}\,P({o}_{t}|{s}_{t})]-\,{\rm{l}}{\rm{n}}\,Z$$

This can be expanded to give:

10 $${\rm{l}}{\rm{n}}\,{Q}^{\ast }({s}_{t}|\pi )={E}_{Q({s}_{t-1}|\pi )}[{\rm{l}}{\rm{n}}\,P({s}_{t}|{s}_{t-1},\pi )]+{E}_{Q({s}_{t+1}|\pi )}[{\rm{l}}{\rm{n}}\,P({s}_{t+1}|{s}_{t},\pi )]+\,{\rm{l}}{\rm{n}}\,P({o}_{t}|{s}_{t})-\,{\rm{l}}{\rm{n}}\,Z$$

Note that the probabilities here can be expressed using only the **A** and **B** matrices. In order to express this as a gradient descent, the difference (${\varepsilon }_{\pi ,t}$) between the current belief about *s*_*t*_ *|π*, and the distribution above can be created, and the temporal derivative of the current belief set equal to (some function of) this difference. Effectively, this corresponds to a gradient descent on free energy such that when the error is zero, expected states cease to change and free energy has been minimised.

This scheme can be used to derive belief updates for each factor of the approximate posterior, and used to map out the connections (i.e., message passing) between different populations of neurons encoding the unknown variables (i.e., hidden states and policies). The results of this mapping will be illustrated for a hierarchical scheme below, in terms of neuronal populations encoding expectations and errors. Note that the estimation of states *per se* requires taking the probabilities of policies into account. This is done by performing a Bayesian model average, where each policy is treated as a competing model, with a different probability of being the correct policy. The model average for states takes the form:

11 $$Q({s}_{t})=\sum _{\pi }Q(\pi )Q({s}_{t}| \pi )$$

As intimated above, in the discussion of their anatomy, the basal ganglia appear to have a clear role in evaluating policies, so it seems likely that their output – via the thalamus to the cortex – could have an important role in this model averaging. As a policy is a sequence of actions, the process outlined above can be used to compute future states based on beliefs about the policy, defining a trajectory through state space. Although the anatomy described in the previous section is beginning to emerge in this discussion of computational architectures, the important hierarchical structure of the cortex and cortico-subcortical loops has yet to be accounted for. We try to address this next, to establish a more comprehensive account of the computational anatomy implied by these sorts of models.

### Hierarchical composition of MDPs

A recent advance in active inference models is the use of a hierarchical MDP as a generative model (see Fig. 4, right)^11^. The addition of hierarchical levels allows for a rich representation of deep temporal structure, in which higher levels of the model evolve over a time scale that is slower than that of the level below. To illustrate this point, the time spent reading each word in this sentence is shorter than that for the sentence as a whole. Thus the states representing individual words would be represented at a lower hierarchical level than the states representing sentences (themselves lower than those representing a page, and so on). In defining this type of structure, it is necessary to define the **A**,**B**,**C** and **D** matrices for each level. In this setting, the probabilities of policies at the lower level become conditioned on the state at the level above, as do the probabilities of the initial states at the lower level.

**Figure 5:**
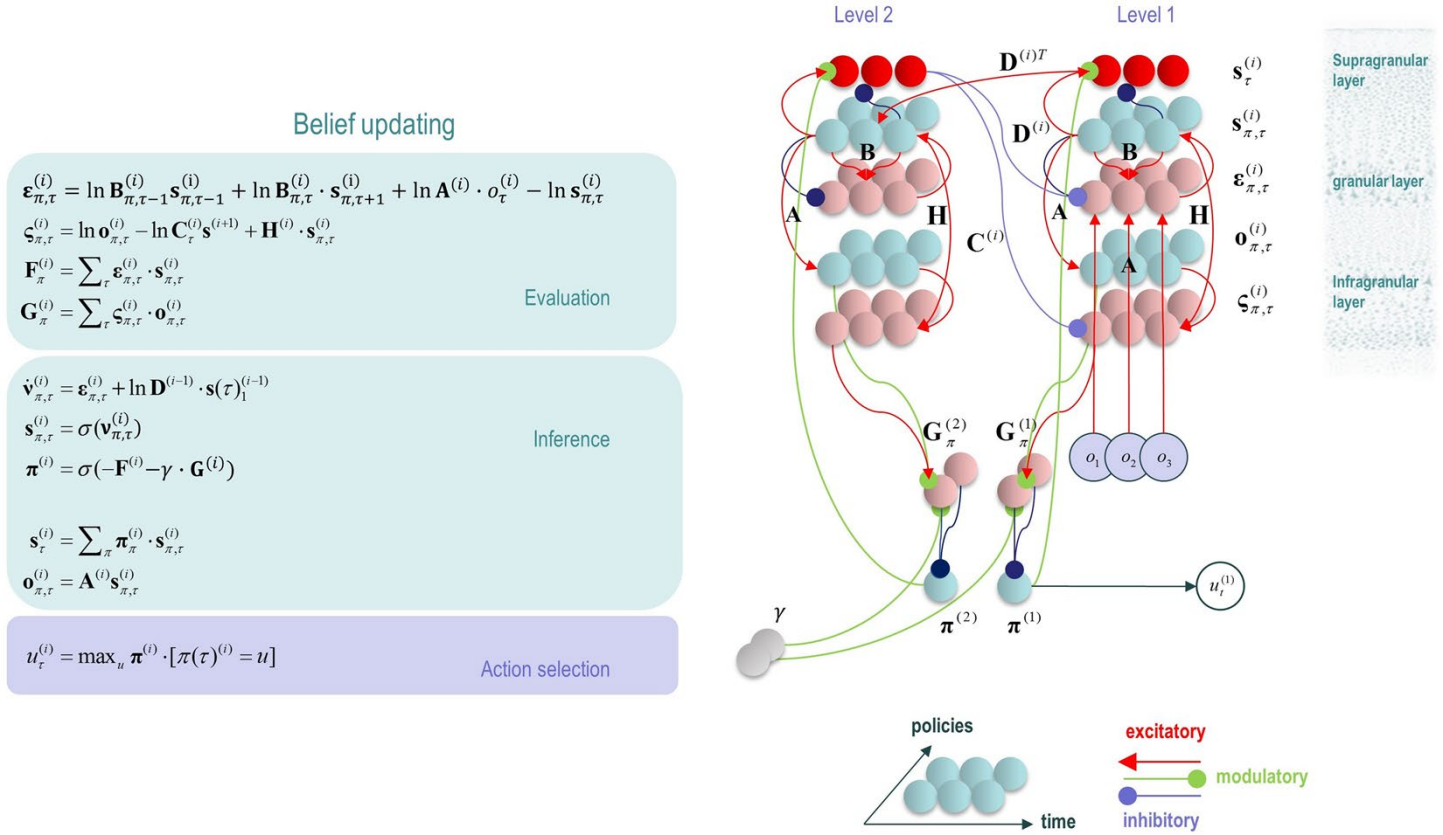
Neuronal message passing for a hierarchical MDP The belief updates are shown *on the left* The first two lines define error variables for states and outcomes. The former is equivalent to the definition of ${\varepsilon }_{\pi ,t}$ given in the text, but here is expressed in terms of the matrices and vectors of beliefs about states. The outcome error term is used to compute the expected free energy. The first two lines in the ‘inference’ box describe a gradient descent to optimise beliefs about ${s}_{\tau }^{(i)}| {\pi }^{(i)}$. To understand this intuitively, consider when the belief $Q({s}_{\tau }^{(i)}| {\pi }^{(i)})$ represents too high a probability for a given value of ${s}_{\tau }^{(i)}$. This results in a negative ${\varepsilon }_{\pi ,t}^{(i)}$, meaning the corresponding component of ${\nu }_{\pi ,t}^{(i)}$ will decrease with time. As the belief $Q({s}_{\tau }^{(i)}| {\pi }^{(i)})$ is a function of ${\nu }_{\pi ,t}^{(i)}$, this will change the beliefs such that this value of ${s}_{\tau }^{(i)}$, conditioned on *π* ^(*i*)^, becomes less probable. This is a negative feedback system, examples of which are ubiquitous throughout biology. The evaluation of policies in the third line of the inference box is similar in form to the prior belief that policies will minimise expected free energy. The inclusion of the free energy is due to the fact that this is an attempt to approximate a posterior distribution, and once an observation has been made, the expected free energy at the previous time step becomes the sum of the free energy and the expected free energy. The fourth line is the Bayesian model average described in the main text. The action selection box provides the system with classical motor reflexes, as they ensure action fulfils expectations about outcomes. *The graphic on the right* provides a graphical (neural network) representation of these equations, where each unit represents one of the variables on the left. The resulting connections are strikingly consistent with a series of hierarchically arranged cortical columns, each of which participates in a cortico-subcortical loop (see Fig. 3). That the subcortical structures in this scheme have the role of evaluating policies is consistent with the known functional anatomy of the cortex and basal ganglia.

To make this more explicit, to go from the basic MDP model described above to the hierarchical form, *P*(*π*) is replaced with $P({\pi }^{(i)}| {s}_{t}^{(i+1)})$, and $P({s}_{1})$ with $P({s}_{1}^{(i)}|{s}_{t}^{(i+1)})$. To build some intuition for this, we again appeal to the hierarchy involved in reading. On selecting a sentence to read, we start at the first word, with the intention of reading the subsequent words. In doing so, our initial state has been determined by the higher level, as has the sequence of actions we will take before moving on to the next sentence. In the description of the basic, non-hierarchical, model above; the principles used to determine belief updates and neuronal message passing were illustrated. Figure 5 shows the results of applying exactly the same principles to the hierarchical model, in terms of the belief update equations, and the anatomy of the neuronal message passing they imply. The computational anatomy that emerges very closely resembles that shown in Figs 2 and 3, with a series of hierarchically arranged cortical columns, connected by ascending and descending patterns of laminar connectivity. In addition, there are loops which correspond to cortico-subcortical loops, which evaluate policies, and include an excitatory cortical input, followed by an inhibition of the output. This connectivity matches that of the direct pathway through the basal ganglia closely, suggesting that expected free energy could be computed by D1 receptor expressing MSNs in the striatum. The units sending the *γ* signal have a connectivity consistent with midbrain dopaminergic regions, and previous work has argued that these regions may signal precision over policies^88^. The update equations for *γ* can be found in^8^.

### Working memory and attention in active inference

The MDP scheme outlined above provides a general but unambiguous formulation of behaviour in terms of an anatomically plausible message passing scheme. This means that it is possible to describe cognitive phenomena in terms of the beliefs a creature has about the world, and their biological manifestation. Taking this approach, working memory can be conceptualised as a process of evidence accumulation in a temporally structured hierarchy. Overt or covert visual sampling of the environment is a serial process^90^, and working memory ensures evidence is accumulated rather than simply acquired. The link between working memory and action posed by some authors^56,91^ manifests in this scheme in two ways. First, the ongoing representations of beliefs about hidden states are influenced by model averaging over policies, highlighting a role for the basal ganglia in influencing working memory representations. Secondly, evidence accumulation over each time scale informs future policies, and is crucial for planning action in a temporally evolving system. In the following simulations, we will show that this approach to understanding working memory is consistent with many of its behavioural, psychophysical, and electrophysiological manifestations.

As hinted at earlier, attention has been used to mean very different things. These broadly fall into two categories: salience and gain modulation. Gain modulation based accounts suggest that attention involves increasing the gain of a particular sensory channel^92^, enhancing the processing of that sensation. This form of attention is most simply addressed in the context of predictive coding^7^, where beliefs about the precision of sensory input weight ascending prediction errors that induce belief updating at higher levels. Although there will be brief discussion of this form of attention later, the main focus of the following will be salience based accounts of attention.

These accounts are very well suited to consideration within an MDP framework, as they describe attention in terms of salience^93–95^, a property of motor planning. If attention is drawn to a salient location, either by an overt saccade or covertly, this corresponds to a behaviourally salient action. The evaluation of policies rests on the expected free energy under each policy, as can be seen in Fig. 5, and this informs an agent’s beliefs, under their generative model, about which policy (e.g., saccadic eye movement) they will pursue. The negative expected free energy corresponds well to the notion of salience and, as was shown earlier, can be split into an explorative and an exploitative component. At low hierarchical levels defining saccadic eye-movements, it seems likely that it is the explorative (uncertainty reducing) component which dominates. This is because the prior probability (preferences or costs) of outcomes – on which the exploitative component depends – is more naturally represented at higher levels. In the context of active inference, a salient location corresponds to the location which, if a saccade were performed to it, would most reduce uncertainty about the state of the world. In short, the uncertainty reducing epistemic part of expected free energy is exactly the expected Bayesian surprise that underlies salience. In this sense, salience becomes an attribute of how we sample the world that offers epistemic affordances – and enables us to forage for information.

## Delay period task simulation

In order to demonstrate that the characterisation above has face validity as a computational account of working memory and attention, we will try to show that associated behavioural and experimental findings can be reproduced by simulating hierarchical evidence accumulation and action to reduce uncertainty. The following simulations additionally provide a concrete example to make the notions of hierarchical processing and salient eye-movements more tangible and explicit.

During our simulated task the subject is, following a fixation period, presented with two scenes one after the other. Each scene consists of pictures of two objects; a cat, a bird, or some seeds. Next, a retrocue is presented. This indicates which of the two scenes the agent is most likely to be tested on at the end of the trial. This is followed by a delay period, which is followed by a probe scene. On presentation of the probe, the agent indicates whether this was one of the two scenes that were initially presented. Successful performance on this task would indicate the maintenance of a representation of the first two scenes during a delay period. The retrocue is included to demonstrate that this working ‘memory’ of the scenes can be manipulated by cues presented after they have been removed. In other words, the past can be reinterpreted in the context of current stimuli. Tasks of this form have been used in numerous experiments with human participants^96–100^.

### The generative model

**Figure 6:**
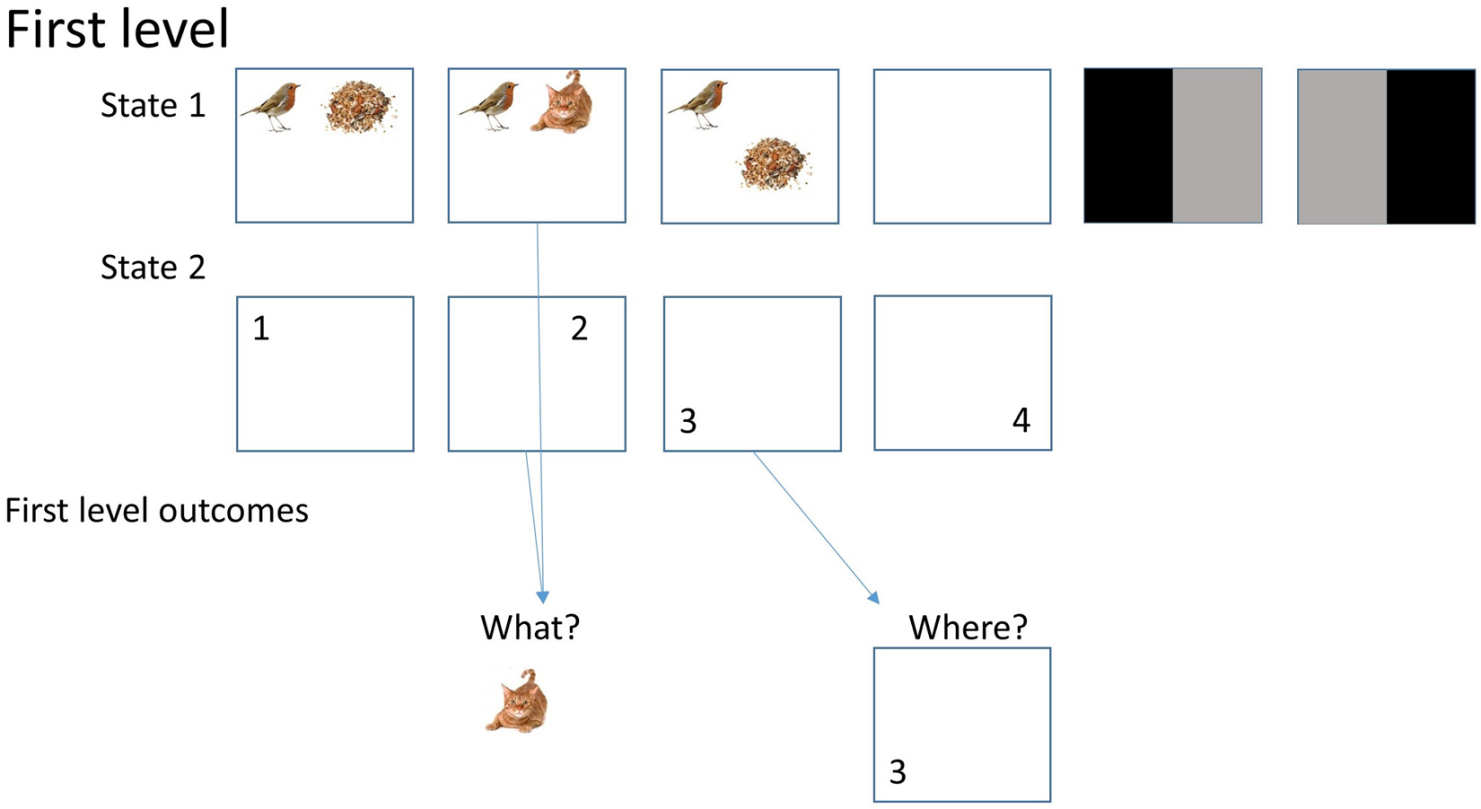
First level of the delay period task generative model: the first hidden state (State 1) is the current scene being presented, which can be any of the scenes shown in the upper row. The first three scenes are the possible scenes for both initial stimuli, and for probes at the end of the trial. The fourth is used for initial fixation and delay period, and the final two are retrocues. The first retrocue indicates that the first scene is likely to be the one they are tested on (although is uninformative about whether the probe will be in the remembered scenes set or not). The second indicates the probe is more likely to be the second scene. Unless stated otherwise, the retrocues had a validity of 90%. The second hidden state (State 2) is the current fixation location. The agent can look to any of the four quadrants in the visual scene, and controls this state via its associated **B**-matrix. The outcomes simply include a visual and a proprioceptive outcome. The arrows show an example of the outcomes that would be generated, according to the **A**-matrix: looking at the second location, when the first state is the second scene will generate the visual stimulus (outcome) of the cat. The proprioceptive outcome is simply mapped from the second state by an identity **A**-matrix (in the relevant dimensions). The stimulus images used here are reproduced from Mirza *et al*. 2016^9^, with kind permission from the authors. https://creativecommons.org/licenses/by/4.0/. No changes have been made to the individual images.

The first (lowest) level of the generative model is shown in Fig. 6. At this level, there is a mapping from two hidden states to two observable outcome modalities. The first hidden state represents the scene that is generating (exteroceptive) visual input, and the second represents the (proprioceptive) position of the eyes. Each scene comprises two images placed at two of four possible locations (i.e., quadrants). The four possible eye positions correspond to foveation of these same four regions in the visual scene. Sensory outcomes include visual or proprioceptive feedback. The probabilities of each outcome conditioned on these states are defined such that the hidden state defining eye position has a direct deterministic mapping to the proprioceptive feedback for each position. The combination of hidden states for both eye position and the current scene define the visual outcome, which is of the images within the scene. This will be whichever image is in the location of the scene which corresponds to the current eye position.

**Figure 7:**
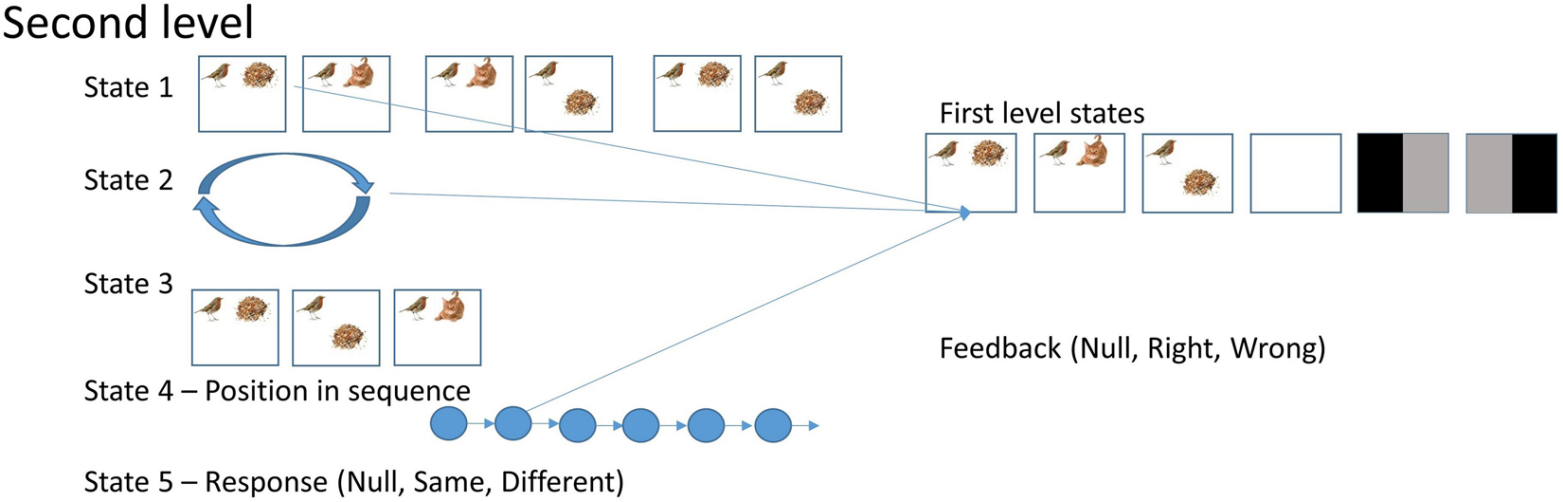
Second level of delay period task generative model This level defines the temporal structure of the task, and selects the scenes (first level state 1 – see Fig. 6). State 1 at this level defines which two scenes will be presented at the start of the trial. State 2 indicates the order in which they will be presented. State 3 is the probe scene presented at the end. State 4 provides an index for the position in the temporal sequence of the trial – this hidden state increases deterministically with time. State 5 is the only state at this level that the agent has control over. It represents the report the agent makes, when presented with the probe. An example of a scene being generated as a lower level hidden state is indicated by the arrows. An outcome at this level provides feedback on the response. The agent expects to be right, and this is expressed in the model as a prior over the feedback outcomes. The stimulus images used here are reproduced from Mirza *et al*. 2016^9^, with kind permission from the authors. https://creativecommons.org/licenses/by/4.0/. No changes have been made to the individual images.

**Figure 8:**
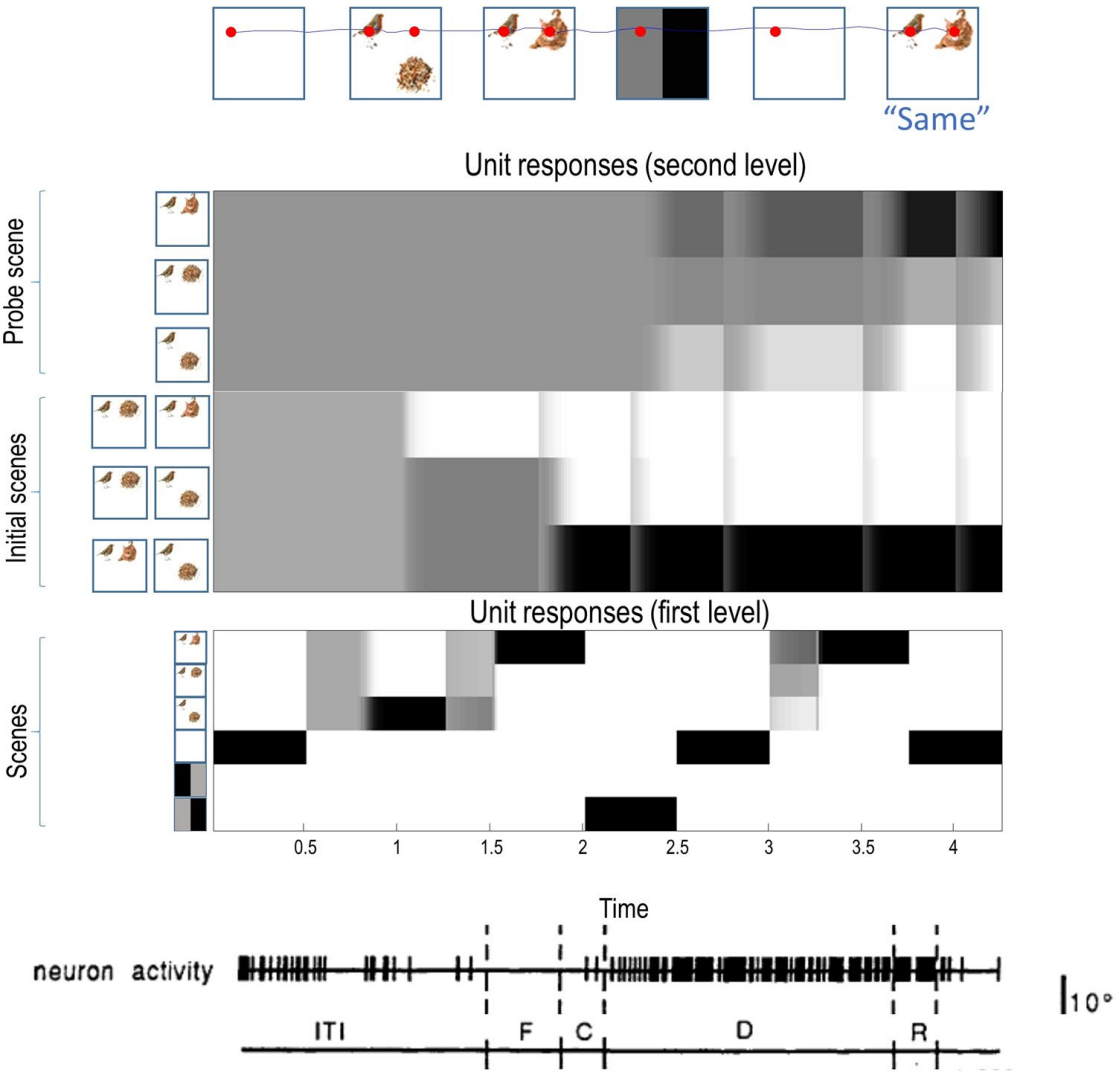
An example trial This illustrates the course of one trial, with a response made at the end. Simulated saccades are shown in the *upper panel*, with fixations indicated by the red dots. Following an initial blank scene, the agent is presented with two scenes in sequence. The subsequent retrocue indicates that the second scene is more likely than the first to be the probe scene. After a delay the probe scene is presented and the agent responds correctly that it is the same as one of the initial scenes. The number of saccades within each scene presentation varies, depending on how many it takes to resolve uncertainty about the scene. Two saccades are sufficient for each scene as, once the agent knows the bird is in the top left, the top right corner should provide all of the necessary information to distinguish between the scenes. The simulated unit responses are shown in the *middle panels*. These represent the sufficient statistics of the approximate posterior beliefs, and are shown for the units representing states 1 and 3 at the second level, and those representing state 1 at the first level (please refer to Figs 6 and 7 for descriptions of these hidden states). Darker shades indicate a greater firing rate. The evolution of beliefs about states is much faster at the first level, and it is easy to match the responses here to the progression of the trial. At the second level, units representing beliefs about the initial scenes start with uniform activity across all three possibilities. On presentation of the first scene, the unit which does not include a representation of it reduces its activity, and the others increase. On presentation of the second scene, the combination of initial scenes is unambiguously inferred. The response of the relevant unit persists throughout the trial. It is this which should be compared to measured neuronal responses^48^ during an oculomotor delay period task, shown in the *lower panel*. In this experiment, the inter-trial interval (ITI) is shown, followed by fixation (F), a cue (C), a delay period (D), and a response (R). The increase in neuronal activity during the delay period matches that of the maintained representation of beliefs about the initial scenes in the simulation. Note that the upper panel is not synchronised to the unit responses, as the number of time steps the agent spends looking at each scene varies. In contrast, the unit responses are synchronised to one another, as is made clear by the vertical grey lines induced by the presentation of a new scene. The stimulus images used here are reproduced from Mirza *et al*. 2016^9^, with kind permission from the authors. https://creativecommons.org/licenses/by/4.0/. No changes have been made to the individual images.

The second level of the generative model, shown in Fig. 7, defines the task itself. It has five hidden states which combine to influence the hidden states at the lower level. The first of these hidden states defines which scenes will be presented at the start of the trial, which the agent will have to remember to respond to the probe at the end. The hidden state can take on three possible values, each of which represents two out of the three possible scenes the agent can be presented with. The order in which these two are presented is determined by the second hidden state. The probe stimulus at the end of the trial is one of the three scenes, and hidden state 3 specifies which. The fourth state provides temporal structure to the trial, and takes on the value of the position in the sequence. During a trial, the sequence begins with a delay, followed by presentation of two scenes, one after the other, a retrocue, a further delay, and then presentation of the probe stimulus. The ensuing trial sequence is shown in Fig. 8. The final, fifth, hidden state is the response the agent makes. This is under the control of the agent, who can decide to declare the probe stimulus the same as one of the scenes at the start of the trial, different to both of them, or to withhold a response. The outcomes at the second level include the first hidden state of the first level, and feedback on the decision the agent has made concerning the probe. The prior beliefs of the agent concerning feedback (i.e., preferences or costs included in the corresponding values of **C** over outcomes) are that it is most likely to be correct, and least likely to be incorrect. According to the exploitative part of the expected free energy equation, the agent will act to fulfil these beliefs.

### Working memory performance

This simulation exhibits some of the characteristic features of working memory. It is able to maintain a representation of initial stimuli presented to it, and to use these representations to inform a future response to a probe stimulus. This information is maintained even during a delay period, where there is no relevant sensory stimulation. Further to this, the remembered information, combined with a retrocue, can be used to manipulate representations of the expected probe item. The representations over time can be seen in the simulated unit responses in the middle panels of Fig. 8, which resemble the activity of neurons recorded in monkeys performing delay period memory tasks^48^. It can be seen in these that, when the retrocue is presented, the beliefs about the probe scene are altered, despite the fact that the retrocue alone carries no information about scenes. This demonstrates that new information about past stimuli can be combined with the memory to make predictions about the future. It can be seen that these beliefs about the future are used to bias evidence accumulation at the lower level as, at time step 3, the agent starts with a belief about the relative probabilities of each scene coming up. That the correct scene is inferred to be most likely demonstrates that the active inference scheme here has successfully solved the Monty Hall problem^101^, and has recognised that, if there are three possible scenes, there is a two thirds chance of the probe scene being one of the scenes initially shown. The retrocue serves to distinguish which of these is more likely.

### Reaction times

**Figure 9:**
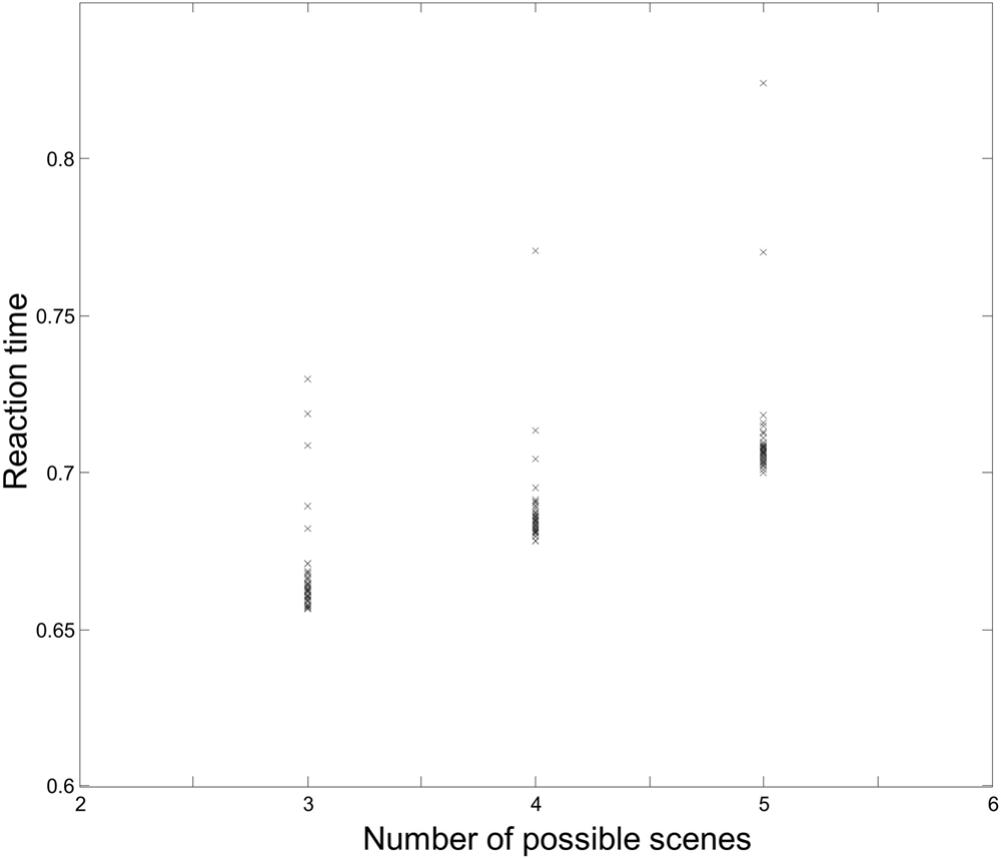
Changes in reaction time when the full set of possible scenes is 3, 4, or 5 Here, the simulation was run 150 times; 50 times for each number of possible scenes. In each case the actual sequence was identical, with a probe scene which was different to both initial scenes. However, the total number of scenes from which the scenes presented were drawn was varied. The reaction time is the computational time taken from the probe stimulus presentation to the response (i.e. the time taken by the computer to simulate this step).

In addition to successful performance of a delay period working memory task, this model reproduces experimental findings from humans performing similar tasks. For example, an analysis of studies using working memory tasks found a linear relationship between response time, and the number of items required to be remembered^102^. To demonstrate that such linear increases in reaction time can result from increasing demands on belief updating, the delay period task from above was simulated with differing numbers of scenes in the total set of scenes. In these simulations, the number of scenes shown at the start of the trial was still two, and the task was still to indicate whether a single probe scene was the same as one of these. However, the generative model allowed for a total of three, four, or five scenes to be selected from. This meant that, if the probe scene was not one of the initial scenes shown, there were a greater number of possibilities to eliminate. For each number of possible scenes, fifty simulations were run, in which the probe scene was different to the remembered scenes. The reaction times for each set of simulations are shown in Fig. 9. These were the measured computational times from the probe presentation until the response. It is clear that this model replicates the linear increases in reaction time found in the experimental literature.

### Evoked responses: Context updating

In addition to behavioural measures as above, a challenge for this simulation is to replicate electrophysiological findings. The first of these electrophysiological simulations was motivated by a study examining the correlates of context updating in the evoked response potential (ERP)^103^. Participants were shown a series of letters, serially presented, and were asked to make responses to particular letters. The required response to the letters changed throughout the trial. If an “A” was presented, a right button press was required for any “X” which followed, and a left button press was required for any “Y”. If a “B” was shown, the required responses were inverted for subsequent letters. This meant that “A” and “B”, while not requiring response themselves, define a change in context for future responses. Comparing ERPs in response to these context dependent stimuli to those for context independent stimuli, the authors described the ERP components that were sensitive to contextual updating. Over frontal electrodes, they identified an early effect of context updating, which was later followed by a prolonged context sensitive response. A later study used functional magnetic resonance imaging to localise the context sensitive frontal region which was active during the time course of the earlier response^104^. The right dorsolateral prefrontal cortex was shown to have a greater response during this time period when a stimulus had context dependency, consistent with the implication of this region in working memory tasks in primate experiments^105^. Note that this region is higher in descriptions of cortical hierarchies than the sensory cortices which might represent the visual scenes in the delay period task simulated above.

**Figure 10:**
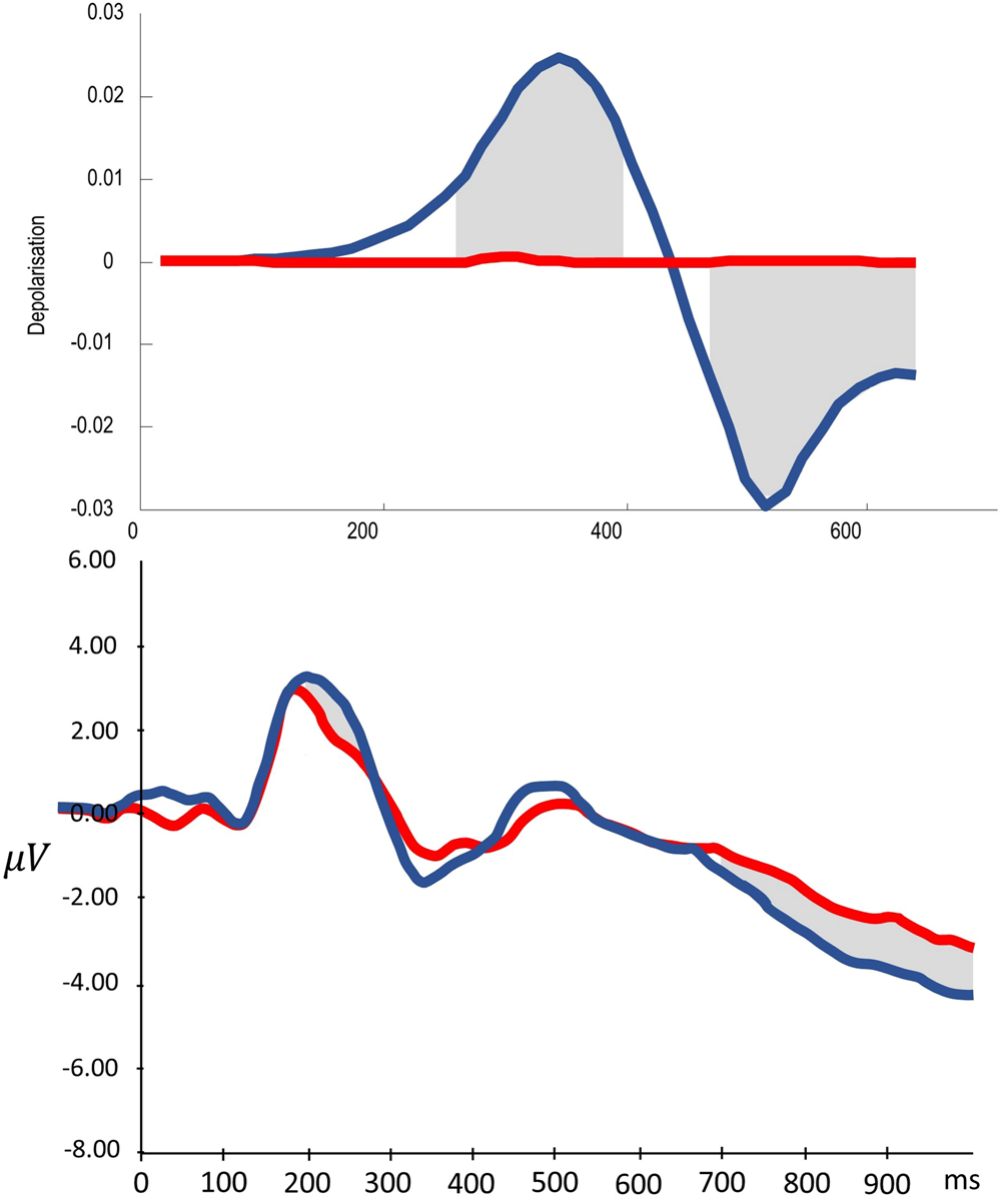
Context dependent components of the ERP The effects of changes in context on the (simulated and real) ERP are shown here. Simulated ERPs are computed from the rate of change of neuronal activity (as shown in the unit response plots in Fig. 8). In other words, they are the $\dot{\nu }$ terms from the equations in Fig. 5, which simulate fluctuations in neuronal depolarisation. In order to test these effects in the simulation, the retrocue validity (in terms of the agent’s belief about how informative it is) was altered. A valid retrocue indicated the correct of the first two scenes with 90% validity, whereas an invalid retrocue had 50% validity. Clearly this is uninformative when indicating one of two options. A valid retrocue therefore defines a change in context, and the ERP simulated in this context dependent situation is shown on the *upper plot* in blue. The same plot shows a red line for context independent cueing, simulated with an invalid (uninformative) retrocue. In both cases, the ERP is generated from the higher level units representing the possible probes (the first units shown in Fig. 8). Note that there is an early context sensitive difference between conditions, which then reverses later on. While not exactly the same timing as in measured ERPs from frontal electrodes, shown in the *lower* plot^103^, the overall pattern of a difference which then reverses later on is remarkably consistent. Homologous differences in ERP responses are highlighted by the shaded grey areas (these were regions of significant difference in the empirical data). Context dependent conditions are in blue, and context independent in red, as in the simulated plots.

In order to see whether the model presented here could reproduce this electrophysiology, the retrocue validity was altered. A high validity would indicate that, for the majority of trials when the probe scene is within the remembered set, the probe scene matches the remembered scene in the position indicated by the retrocue. Low validity would mean that, for trials where the probe is in the remembered set, the retrocue is uninformative about which remembered stimulus is likely to be a match. This resembles the ERP study described above, as a valid retrocue corresponds well to a context dependent stimulus, whereas an uninformative retrocue should cause no updating of context (i.e., working memory). As noted above on the basis of the anatomy, in addition to the notion that a slowly evolving context should be represented at a higher hierarchical level, the ERPs of interest are likely to be those in the higher of the two levels of interest in the simulation. Simulated ERPs^106^ from the higher level, corresponding to the units representing the final probe, are shown in Fig. 10. As can be seen clearly in the difference between waveforms, there is an early difference, which is later inverted. This resembles the results of^103^ in frontal electrodes.

### Evoked responses: Load dependency

**Figure 11:**
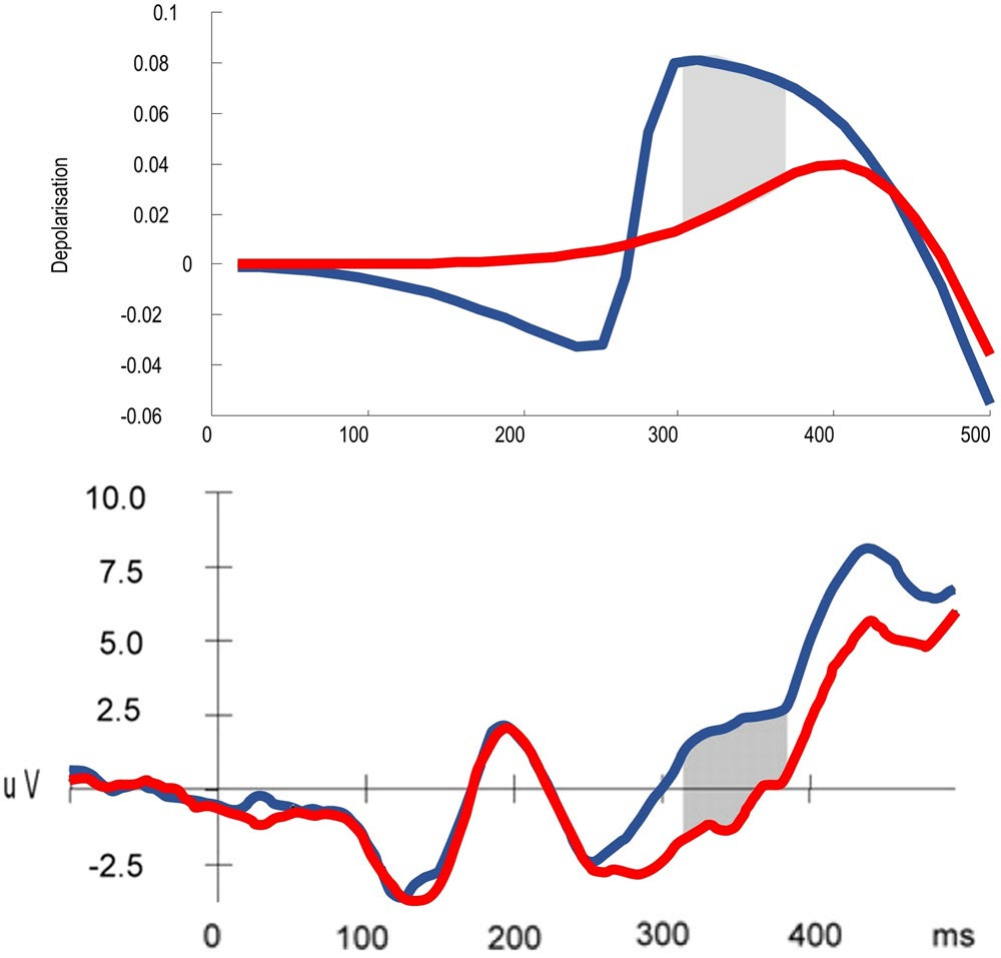
ERP dependence on memory load The simulated ERPs shown in the *upper plot* are based on the same valid (blue) and invalid (red) retrocues as in Fig. 10. However, here we show the responses to the probe stimulus rather than the retrocue itself. There is an obvious difference between the two conditions which starts at around 300ms, highlighted in grey. In the experimental results in the *lower plot* ^107^ a difference is shown in this region in different memory load conditions. The load conditions refer to how many items are remembered before the subject is tested on a probe. This load dependence has been shown to be abolished by retrocues^108^, and the change in the simulated ERPs is consistent with this abolition, when moving from the invalid to valid condition.

The N3RS (“Retroactive search”) is an ERP component which has been reported to be sensitive to working memory load^107^. It is found in the evoked response following probe presentation. Interestingly, it can be abolished by retrocues^108^. This makes intuitive sense, as a valid retrocue tells the participant that they only need retain one of the items they initially remembered. The effective load by the time the probe is presented should then be simply one item, regardless of the actual number of items initially memorised. Figure 11 shows the simulated ERP following probe presentation in the conditions in which a retrocue is valid or uninformative. An uninformative retrocue should replicate the N3RS, which should be abolished by the valid retrocue, which confers a lower expected load. The simulated ERP taken from the unit representing the probe item show a clear and sustained difference which is remarkably consistent with the abolition of an N3RS response.

In summary, the simulations illustrate the intimate relationship between working memory and salience in driving evidence accumulation that realises the epistemic affordance of saccadic targets. Based upon the normative principles of active inference, we were able to reproduce fairly complicated electrophysiological responses (and reaction times), in terms of contextual updating and load dependency. In brief, valid retrocues augment ERP components, relative to uninformative invalid cues that do not require any (belief) updating about the remembered context. They additionally abolish load dependent effects to probe stimuli, in virtue of their ability to discount the number of possible outcomes. Given that load dependent effects decrease the amplitude of ERP components, the abolition of these manifests as an augmentation of these components.

From the perspective of active inference, these effects are almost self-evident: belief updating about the prospective probability of a probe stimulus can only be induced if the retrocue reduces uncertainty (i.e., if the retrocue is valid). Subsequent belief updating, on presentation of the probe stimulus, can then proceed more rapidly, with self reinforcing and self consistent messages from the past (retrocue) and present (pro). This increase in the rate at which evidence has accumulated is reflected in the amplitude of the simulated ERP. These subtle but sensible aspects of evidence accumulation rest on sampling informative sensory evidence and accumulating that evidence in a Bayes optimal fashion using biologically plausible belief propagation schemes. Crucially, these sorts of behaviours can only be realised with a generative model that explicitly represents the past and future; in other words, a generative model that is equipped with a working memory.

### Dopamine

**Figure 12:**
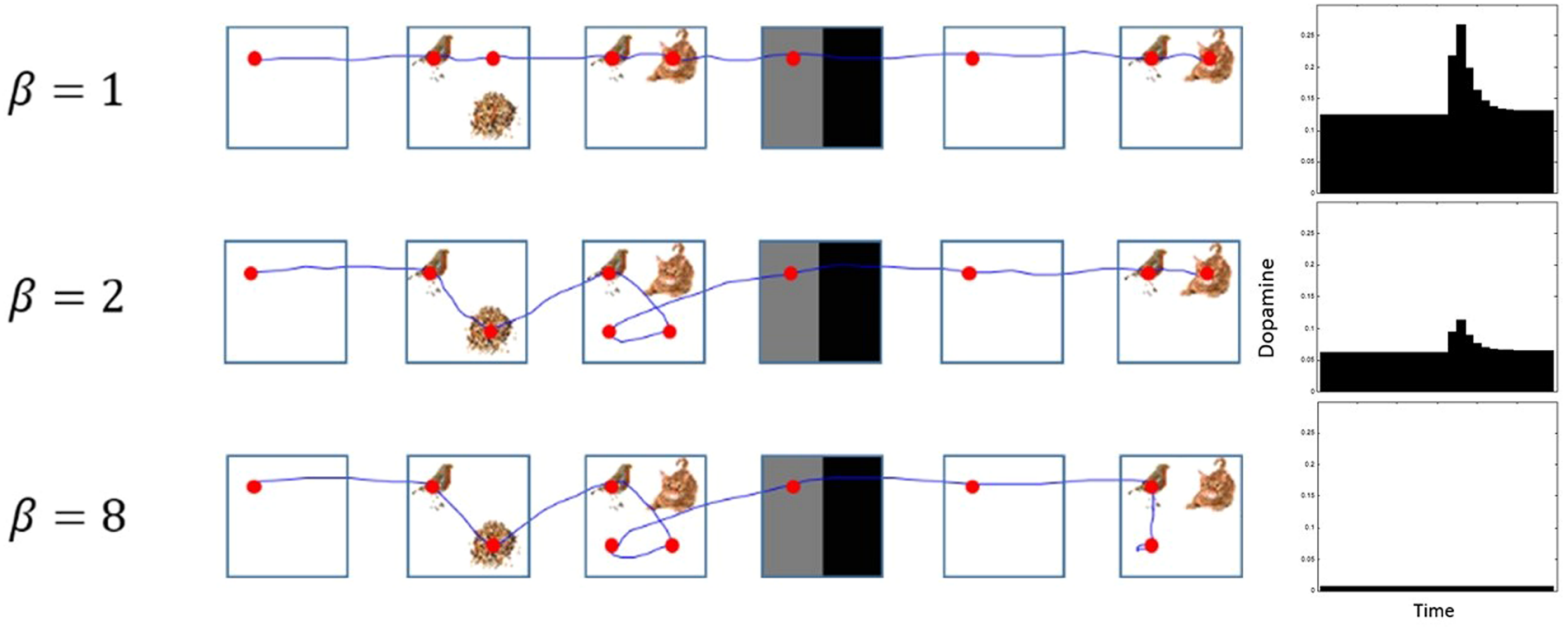
Effects of dopamine modulation on salience Here each row shows the same trial, but with decreases in *γ* (*β* is the inverse of *γ*) relative to the row above, corresponding to a reduced dopaminergic response, and reduced precision of beliefs about policies. See the main text for a discussion of these behaviours. The plot on the right shows a simulated phasic dopamine burst in response to the probe stimulus at the end of the trial (at the second level of the model). The same response is shown for each value of *β*, to demonstrate the attenuation of the signal with decreasing *γ*. The stimulus images used here are reproduced from Mirza *et al*. 2016^9^, with kind permission from the authors. https://creativecommons.org/licenses/by/4.0/. No changes have been made to the individual images.

As noted in the description of the neuroanatomy of saccadic eye-movements above, dopaminergic projections to the striatum appear well placed to influence the control of saccades, and disruption of these projections disrupts normal patterns of eye movements^29^. In order to interrogate this in the MDP scheme we introduced an inverse temperature parameter *γ* to moderate prior beliefs $P(\pi )=\sigma (-\gamma \cdot G(\pi ))$. This is shown in Fig. 5. Using exactly the same formalism as above, belief updates about this sensitivity or precision parameter can be derived and simulated^109^. We have previously shown that the behaviour of this parameter reproduces many of the characteristics of phasic dopamine responses^110–112^. This suggests that dopamine responses may report the precision of beliefs about – or posterior confidence in – policies. The plot on the right of Fig. 12 illustrates the relationship between simulated dopamine and precision in response to the probe (at the second level of the model). By reducing the prior expectation of this parameter to very low levels, the simulation acts as if it had received a bilateral MPTP infusion to its caudate nuclei, or as if it had a Parkinsonian syndrome.

As can be seen in Fig. 12 the simulation with the highest *γ* (lowest $\beta =\frac{1}{\gamma }$), in the top row, produces an optimal sequence of saccades. Two fixations are always sufficient to interpret the scene. In the second row, the first clear effect (of simulated dopaminergic deficits) is a choice to look to the lower right when the first scene was presented. The lower right quadrant is a salient location, as it allows the only diagonally arranged scene to be distinguished from the other two, horizontally arranged, scenes. However, it is not as salient as the upper right quadrant, as the absence of a stimulus here would confirm it was the diagonal scene, but also allows the agent to distinguish between the two horizontal scenes. The lower right quadrant does not provide sufficient information to identify any scene unless there are seeds there. It is this situation the agent finds itself in, when interrogating the second scene. Intuitively, it makes sense that this suboptimal, but partially justifiable, choice occurs with only a small reduction in dopamine. In the second scene in the second row, the failure to resolve uncertainty during the first two fixations prompts an additional saccade. This is suboptimal, as it involves fixating on the only location (lower left quadrant) that is never informative (as it is identical in all three scenes). However, on presentation of the probe, the choices that follow are again optimal. It is plausible that this is influenced by the expected probe scene, as a belief that a diagonal scene is unlikely to be presented entails that the lower right quadrant is unlikely to contain information which might resolve uncertainty. By the third row, with the lowest levels of dopaminergic modulation, even this *post hoc* optimality is lost, as the agent chooses to fixate on the only uninformative quadrant.

This aberrant behaviour can be explained by appealing to the effect of *γ* on the belief updating scheme. It is easiest to see this by considering the extreme values. As *γ* increases, the probabilities of each policy diverge, so that the policy with the lowest expected free energy becomes more likely, and competing policies less likely. As $\gamma \to \infty$, $P(\pi \ast )\to 1$, where $\pi * =\mathop{min\; G(\pi )}\limits_{\pi }$. As *γ* → 0, the opposite happens, and the probabilities of each policy converge. The lower *γ* becomes, the more stochastic policy choice becomes. This explains the suboptimal saccades shown for decreasing *γ*. The increase in number of saccades is also related. In this simulation, the agent moves to the next scene when they have resolved uncertainty. Increasing uncertainty about policies also has an impact on beliefs about states, as the model average of distributions over states involves a smaller contribution from the most likely policies, but a larger contribution from the less likely policies. In conditions of greater uncertainty, more saccades are made in a given scene to try to resolve this uncertainty. These simulated lesions are consistent with the notion that the basal ganglia are crucial for policy selection, here in the context of saccadic policies, and with the importance of dopamine in modulating this function of the basal ganglia^113^. An interesting empirical validation of this would be to ask Parkinsonian patients, on and off dopaminergic medication, to perform this task. We would predict that a model inversion, using their behavioural data, would show a significant and selective between-group difference in the *γ* parameter: see^114^ for a worked example of this sort of empirical testing.

These simulations illustrate two things. First, the consistency with functional anatomy; in terms of the intimate relationship between the dopaminergic projections and the cortico-basal ganglia-thalamic loops that would be predicted if the former mediated the precision of the policy selection associated with the latter. Second, the simulations reveal the importance of encoding precision and uncertainty, for veridical active inference. In this instance, we have been dealing with the precision of beliefs over policies. In the final section, we will consider the precision of beliefs over hidden states of the world in terms of volatility and likelihood precision. It is this precision that we will associate with attention of a perceptual sort.

## Volatility and forgetting

Anticipating an objection to the above modelling of working memory, this section addresses the temporal constraints on working memory in humans. In the above simulations, the higher level representations of the remembered items persist indefinitely once those states have been inferred by the agent. In contrast, behavioural work has shown an increase in the size of errors in delay period tasks with an increasing length of delay between the initial stimulus presentations and the probe item^100^. The accuracy of a maintained representation degrades with time, in a manner not accommodated above. This reflects the beliefs (we have assumed) an agent might have about their environment. In the simulations above, the **B**-matrix, which expresses the transition probabilities $P({s}_{t+1}| {s}_{t},\pi )$, is set to be the identity matrix for both the states representing the remembered stimuli and the probe stimulus. This corresponds to a belief that these states are intransigent and unchanging. In reality, few things in the environment are unchanging, and it might be more reasonable to assume an element of volatility in state transitions. Volatility may be defined in terms of the entropy of the columns of the **B**-matrices:

12 $$H[{{\bf{B}}}_{{s}_{t}=j,\pi }]=-{E}_{P({s}_{t+1}| {s}_{t}=j,\pi )}[\,{\rm{ln}}\,P({s}_{t+1}| {s}_{t}=j,\pi )]$$

**Figure 13:**
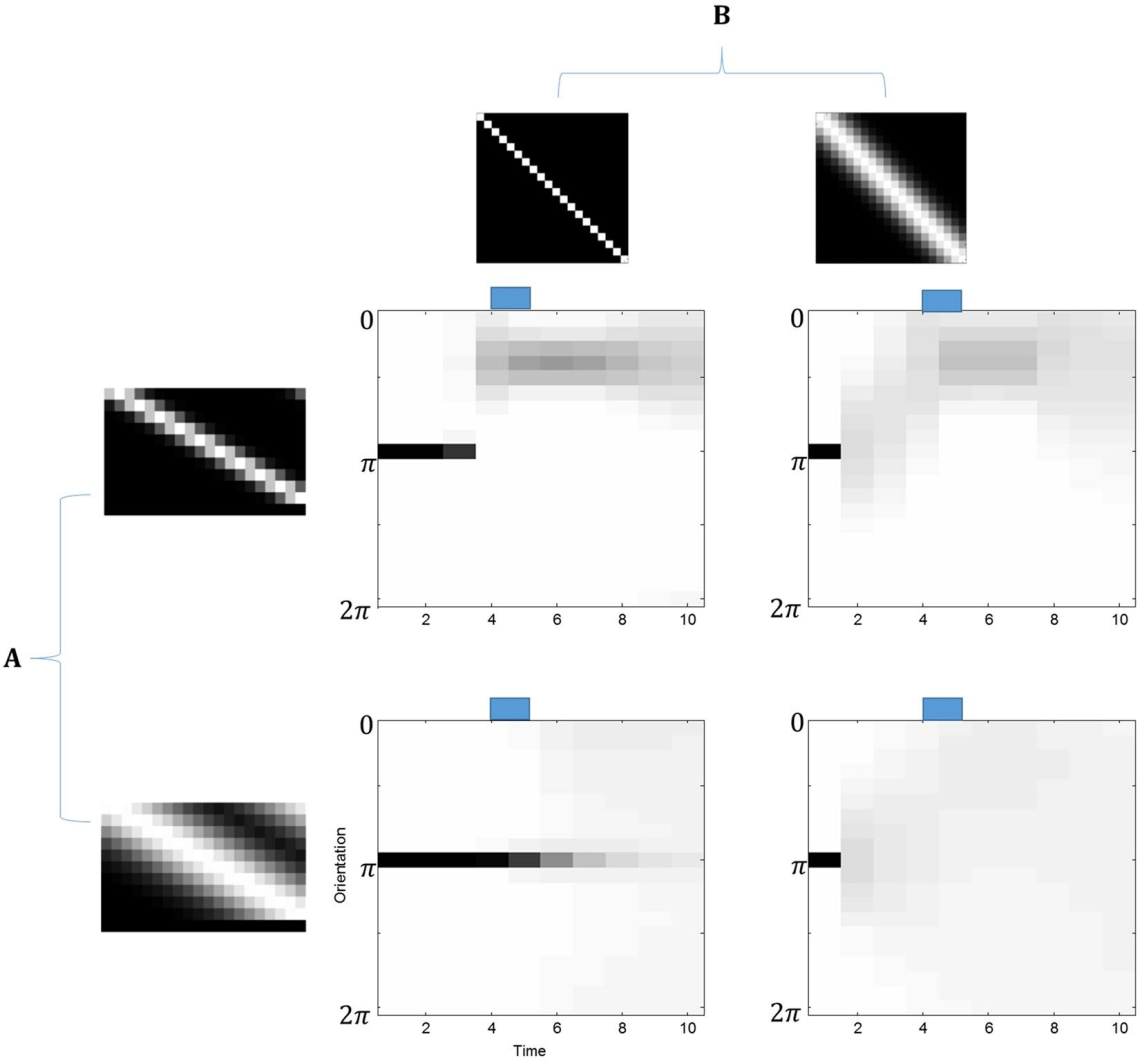
The effects of volatility and observation noise on working memory representations Here a model is used which only has one hierarchical level. One hidden state is orientation, and another is whether the stimulus (e.g. oriented bar) is visible or not. The blue bar above each plot indicates the time for which a stimulus is present. The initial beliefs are set such that the agent believes the orientation is close to *π* radians. From time-step 4 to 5, a visible stimulus is presented at around *π*/2 radians. The **A** and **B**-matrices for each of the four trials have been modified to increase or decrease their precision (or a discrete analogy of this), seen by dispersion around the main diagonal in the graphical representations. The trials in the upper row have a precise **A**-matrix, which corresponds to a high sensory signal to noise, while the lower row has a less precise **A**-matrix. The left column trials have a precise **B**-matrix, meaning small fluctuations in orientation, while the right column trials show much greater volatility with a less precise **B**-matrix. The format of the responses is the same as in the middle panels of Fig. 8 and presents the posterior expectations of the bar’s orientation over 20 orientation bins (i.e., hidden states).

To make this concept more intuitive – and to tie this to the experimental work mentioned above^100^ – it is worth considering the example of a one dimensional state in metric space. If the variable remembered is the orientation of a bar, the state representing this can take on a set of discrete values (in an MDP) representing the possible angles of the bar. If **B** is the identity matrix, the belief about the orientation is that it will remain constant. However, if the matrix components around the main diagonal are allowed to take non-zero values, this means the agent believes orientation is volatile – and can change to neighbouring angles with a finite probability. Volatility does not have to be spatially constrained, but this is assumed here for ease of visualisation. Figure 13 shows a trial in which an agent starts by believing a bar is at a particular orientation. The beliefs of the agent concerning the orientation of the bar are shown throughout the time course of the trial. Comparing the two trials on the bottom row, it is clear that this belief is robustly maintained in the left trial, in which the **B**-matrix defined a very low volatility. In contrast, there is rapid degradation of a clearly defined belief in the right graphic. Here the **B**-matrix defines a high volatility. Biophysical modelling of working memory^115^ has made use of NMDA receptors on inhibitory interneurons to alter a quantity which is equivalent to volatility as defined here.

The simulations here demonstrate that people are likely to have prior beliefs that their environment is volatile. It additionally appears that the more volatile an environment is believed to be; the more quickly its features are forgotten. This is an empirical prediction arising from this formulation. That the **B**-matrices, which determine policy selection, have a potentially important role in determining the robustness of a memory provides an interesting point of contact with work suggesting the basal ganglia might influence working memory in addition to motor policies^57^. This provides an interesting computational link between subcortical processes and the maintenance of working memory representations. This will be addressed in future work. The notion of volatility or precision among state transitions has been introduced here as a prelude to discussing attention; in terms of updating beliefs not about hidden states, but about the parameters of the generative model that are constituted by the likelihood **A** and transition **B** matrices.

## Attention and precision

It is worth reiterating the distinction here between the Markov decision process formulation of neuronal message passing under active inference, and the closely related predictive coding formulation. While the formulation here is defined in terms of a discrete state space, predictive coding relies on a continuous state space. Formulating the scheme here in terms of metric space, as in the discussion of the **B**-matrix above, crudely approximates the representation of continuous variables. This provides an interesting point of contact with predictive coding which informs the distinction between different uses of the word “attention”.

With this aim in mind, it is worth subjecting the **A**-matrix to similar scrutiny to that of the **B**-matrix above. This (likelihood) matrix expresses the probability of a particular observation, conditioned on a hidden state, $P({o}_{t}| {s}_{t})$. In predictive coding, this probability would be represented by a Gaussian distribution, the exponent of which is a precision weighted squared difference between the observed and expected values (the prediction error). Increasing the dispersion of the distribution in this case would correspond to a decrease in precision (i.e., signal-to-noise). It is the optimisation of precision, which therefore controls the gain of prediction errors. This optimisation of precision has been associated with attentional modulation in predictive coding^7^. In fact, it can be shown for any generative model based upon exponential family probability distributions, there is always an update scheme that can be expressed in terms of precision weighted prediction errors; e.g.,^116^. This means that the encoding of uncertainty implicit in the estimation of precision plays a ubiquitous role in hierarchical inference.

Although the MDP scheme here does not pass prediction errors as messages between cortical areas, and does not represent $P({o}_{t}| {s}_{t})$ as a continuous distribution, the entropy of the **A**-matrix can still be optimised so that it has the appropriate precision (i.e., entropy over outcomes, given a particular hidden state). This entropy is defined as for the **B**-matrix above, but does not encode beliefs about volatility. Instead it defines beliefs about the precision of observations. A precise outcome therefore uniquely identifies a particular hidden state – or more exactly, a given hidden state produces one and only one outcome. In contrast, an imprecise likelihood means that the sensory sample of observation is not uniquely determined by its cause. In Fig. 13, the impact of changing beliefs about the likelihood or observation mapping can be seen clearly. The upper row shows trials with the belief that observations are precise, while the lower row shows those where observations are imprecise (e.g., a low signal-to-noise). The columns vary only in the beliefs about volatility. In all trials shown, from 4–5 seconds, a bar is presented with an orientation that differs with respect to that retained in memory (i.e., prior beliefs about the initial state of the world). It is clear that the precision of both the likelihood and transition matrices have a profound effect on evidence accumulation – and that they interact. Of particular interest here is the effect of a precise likelihood mapping that effectively speeds up evidence accumulation (c.f., attentional selection).

Attention defined as modulation of precision, or its analogy in a discrete state space, ensures that more precise data has a greater impact on internal representations. Figure 13 shows that when a stimulus (blue bars) is’attended’ to, it is more likely to update maintained representations, which is here considered to be working memory. If it is not ‘attended’ to, it has little influence over the beliefs about hidden states. It seems from this that working memory updating requires a belief that sensory data is precise, but that maintaining a representation in memory in the presence of distractors requires believing new sensory data is noisy and volatility is low. Another way to conceptualise this is to consider that this modulation biases updating of beliefs (working memory) towards either the current beliefs about the environment, or towards the current incoming data from the sensorium. This fits comfortably with the view that working memory is an attentive process^117,118^, as resistance to distracting stimuli occurs when there is’attentive‘ biasing towards current beliefs, ensuring the posterior beliefs are kept close to prior beliefs.

It is interesting to note that the formulation of beliefs about state transitions and sensory noise in the spatially constrained fashion used in Fig. 13, closely resembles models of working memory as a line attractor^119–121^. There is a large literature making use of continuous attractor models to represent maintained representations of continuous variables, which are updated over time. This includes their use in explaining the network basis for navigation^122,123^, representations of eye position^124^, and motor commands using population vectors^125,126^. This suggests that this kind of representation could be common to many neuronal representations of hidden states that live in a metric space; i.e., mapped representations^127^. There is a sense in which all of these can be considered types of working memory, as all involve a maintained representation that is updated slowly over time by new sensory data. Interestingly, because likelihood and transition precisions are encoded by the model matrices, this means that there (attentional) optimisation corresponds to changes in (neuromodulatory) synaptic connectivity or efficacy – in exactly the same way that attention has been proposed to operate in the context of predictive coding (e.g., through changing the efficacy of self inhibition of pyramidal cells encoding prediction errors).

## Attention and salience

Attention is not always used in the sense of favouring those sensory channels which represent precise information. Descriptions of spatial attention often rely on terms such as “covert” and “overt” attention^94^, which ties the deployment of attention to saccadic eye-movements. The premotor theory of attention^4^ equates overt attention to the foveation of the attended region of space, and covert attention to the planned saccade which would result in foveation of the attended region, if executed. There is an important correspondence between this and the definition of attention in terms of precision, as the high density of photoreceptors in the fovea of the retina ensures that more precise information is obtained from a foveated location in visual space. Active inference implies that saccades will furnish information rich, uncertainty reducing sensations, which are afforded by high-resolution foveal sampling. Despite this link, precision modulation is clearly not the same process as selecting an eye-movement. The latter is entirely dependent on the policy being pursued, whereas the former could occur in the absence of action. In other words, there is a fundamental difference between attention in the context of perceptual inference and state estimation (that depends purely upon the precision of sensory input and beliefs about states of the world) – and salience in the context of active inference (that depends upon the epistemic value of an action). In the language of active inference, the salience of a target in visual space is determined by the agent’s belief about the probability they will make a saccade to that location. This is determined by expected free energy associated with that policy. To emphasise the difference between uses of the word “attention”; we would say that attention is afforded to the causes of sensations while salience is afforded to the way sensations are sampled. Given that the role of working memory is to evaluate future policies, the salience form of attention is inextricably linked to working memory^128^. This could explain the number of attempts to equate the two processes^117,129^, or to consider one a special case of the other^130^.

So-called salience maps have been found in the superior colliculus^131,132^. However, there are a number of ways these could be incorporated into the computationally defined anatomy above (Fig. 5). It could be that the input the superior colliculus receives from the basal ganglia (see Fig. 1) represents the probability of each policy, and this is simply mapped topographically by the superior colliculus. This fails to account for the cortical input. Noting that this cortical input arises from layer V, the same as the laminar origin of cortical input to the basal ganglia^18,36^, it is possible that the messages from cortex to colliculus are outcome prediction errors (${\varsigma }_{\pi ,\tau }^{(i)}$ in Fig. 5) or outcome beliefs (${\varepsilon }_{\pi ,\tau }^{(i)}$) as with the input to the striatum. If so, this would imply a direct role in salience calculations, and would suggest the collicular salience map is the point at which salience (i.e., expected free energy) for each saccade is computed. In this case, the superior colliculus would be playing the role of the basal ganglia, and would be expected to reciprocate output back to the cortical areas from which it receives input. This is the case^133^, as the frontal eye fields receive an input from the colliculus via the thalamus. Just as the first suggestion failed to account for the cortical input, this fails to account for input from the basal ganglia. Other possibilities include that it functions similarly to a cortical column, which might explain its laminar structure, in addition to its communication with both the basal ganglia and cortical regions. This leaves the explanation for its role as a salience map difficult to reconcile. Plausibly, the superior colliculus embodies some combination of these roles.

## Conclusion

The formulation in this paper has demonstrated that important, but often ambiguous, psychological terms can be defined in terms of computational processes performed by the brain. Working memory can be thought of as a process of evidence accumulation in deep temporal hierarchies. This represents the evidence for competing hypotheses about the causes of sensory data. By representing policies as sequences of actions, working memory can be used to predict future states according to the trajectory defined by a given policy. In doing so, it allows for optimal policy selection with respect to future outcomes. Attention has been described in terms of two distinct processes. These are salience and precision attribution. The former underwrites policy selection in, for example, the context of saccadic eye-movements, while the latter relates to beliefs about the precision of sensory evidence relative to prior beliefs about their causes. Clearly salience, on this definition, is intimately tied to working memory. The relationship is consistent with intuition, as the choice of locations to sample should be informed by past sensory samples. Notably, this is a bidirectional influence, as sensory sampling provides evidence for and against the beliefs held in working memory. The link between precision and attention has been touched upon. Precision can have a profound impact on the entry or assimilation of new data into working memory. Conceptualising these processes in this way allow us to describe the relationships between them and the neuroanatomy which supports them. As shown by the simulations above, they are also able to reproduce several empirical phenomena associated with these key processes.

### Data availability statement

The simulations presented in this paper were performed using a standard software routine, **spm_MDP_VB_X.m**. This is available as Matlab code in the SPM academic software: http://www.fil.ion.ucl.ac.uk/spm/
